# Chemical biology reveals CARF as a positive regulator of canonical Wnt signaling by promoting TCF/β-catenin transcriptional activity

**DOI:** 10.1038/celldisc.2017.3

**Published:** 2017-01-31

**Authors:** Xiaoli He, Wenjuan Zhang, Chen Yan, Fen Nie, Chen Li, Xiaofen Liu, Cong Fei, Shengdi Li, Xiaomin Song, Yingying Jia, Rong Zeng, Dianqing Wu, Weijun Pan, Xiaojiang Hao, Lin Li

**Affiliations:** 1 State Key Laboratory of Molecular Biology, CAS Center for Excellence in Molecular Cell Science, Institute of Biochemistry and Cell Biology, Shanghai Institutes for Biological Sciences, Chinese Academy of Sciences, Shanghai, China; 2 Institute of Health Sciences, Shanghai Institutes for Biological Sciences, Chinese Academy of Sciences and Shanghai Jiao Tong University School of Medicine, Shanghai, China; 3 State Key Laboratory of Phytochemistry and Plant Resources in West China, Kunming Institute of Botany, Chinese Academy of Sciences, Kunming, China; 4 Key Laboratory of Systems Biology, Institute of Biochemistry and Cell Biology, Shanghai Institutes for Biological Sciences, Chinese Academy of Sciences, Shanghai, China; 5 Vascular Biology and Therapeutic Program and Department of Pharmacology, Yale School of Medicine, New Haven, CT, USA

**Keywords:** CARF, chemical biology, NC043, small molecule, Wnt signaling

## Abstract

Wnt/β-catenin signaling regulates multiple biological processes and aberration of this pathway is frequently observed in human cancers. Previously, we uncovered NC043 as a small-molecule inhibitor of Wnt/β-catenin signaling. Here, we identified CARF as the cellular target of NC043. We found that NC043 binds directly to CARF through forming a covalent bond with the Cys-516 residue of CARF. Further study revealed that CARF interacts with Dvl, which potentiates the Dvl–c-Jun–β-catenin–TCF transcriptional complex and thus promotes Wnt signaling activation. NC043 could disrupt the interaction between CARF and Dvl, thereby impairing Wnt signal transduction. In line with this, knockdown of CARF in zebrafish leads to impairment of embryonic development, hematopoietic stem cell generation and caudal fin regeneration. Collectively, we identified CARF as the cellular target of NC043 and revealed CARF as a positive regulator of Wnt/β-catenin signal transduction.

## Introduction

Canonical Wnt signaling, also known as Wnt/β-catenin signaling, has critical roles in many physiological processes such as embryonic development and tissue homeostasis. Aberration of this pathway has been linked to a variety of human diseases including many cancers [1, 2]. As an important oncogenic pathway, Wnt/β-catenin signaling has attracted lots of attention and efforts in developing compounds that can modulate this pathway to treat various Wnt-driven human diseases [3–7]. β-catenin accumulation is a major event in canonical Wnt signal transduction. Physiologically, binding of Wnt ligands with their membrane receptors recruits the downstream signal mediator Dishevelled (Dvl/Dsh) to form a signalosome, leading to subsequent release of β-catenin from a complex known as destruction complex—composed of glycogen synthase kinase 3 (GSK3), adenomatous polyposis coli (APC), casein kinase 1 (CK1), Axin and so on [8, 9]. After accumulation, activated β-catenin translocates into the nucleus and forms a complex with the transcriptional factor T-cell factor/ lymphoid enhancer-binding factor 1 (TCF/LEF1) to initiate transcription of Wnt target genes [10].

Dvl is a well-recognized cytoplasmic regulator of Wnt/β-catenin signaling despite of limited understanding so far regarding some details behind Dvl activation and function. In addition to functioning in the cytoplasma, we previously uncovered an important function of Dvl in the nucleus. We found that the nuclear Dvl upregulates β-catenin/TCF4 transcriptional activity through scaffolding a transcriptional complex comprising β-catenin, TCF4 and c-Jun [11]. In recent years, more evidence has been provided to highlight the significant role of nuclear Dvl in Wnt signaling [12–14]. These findings of ours and others indicate that the nuclear pool of Dvl, although constitutes a very limited part of total Dvl, exerts indispensable function in Wnt signaling.

CARF (the collaborator of ARF) was initially identified as a collaborator of ARF in activating p53 [15]. Later studies showed that CARF could interact with p53 directly and facilitate p53 activation independent of ARF [16, 17]. CARF seems to have a fundamental role in controlling cell proliferative fate through both p53-dependent and -independent mechanisms. Overexpression of CARF upregulates p53*/*p21WAF activation and causes premature senescence [18]. On the other hand, CARF could function independently of p53 through modulating ATR/CHK1 pathway, by which CARF silencing induces DNA damage response and eventfully triggers apoptosis [19]. Of note, in p53-deficient cells, CARF overexprssion leads to an opposite proliferative outcome—cell proliferation rather than growth arrest—probably through transcriptional repression of p21WAF1, and activation of E2F1 and MMP. Moreover, CARF overexpression is also shown to promote tumor invasion and metastasis in the context of p53 deficiency [20]. In line with a tumor-promoting effect, CARF silencing is observed to induce suppression of tumor growth in a human tumor xenograft mouse model [19]. These findings clearly suggest the potential of CARF as an oncogenic protein at least in some circumstances such as when p53 function is compromised. So far many details regarding how CARF may facilitate tumor initiation/progression remain largely unknown. In addition to the factors mentioned above, other pathways may be involved in mediating the tumor-promoting function of CARF.

Previously, in an effort to develop drug leads for modulating Wnt/β-catenin pathway, we identified NC043 (15-oxospiramilactone) as the most potent compound from a ~4000-compound library. Moreover, we found that NC043 functions through impairing β-catenin/TCF4 association in an indirect way [3]. To identify the cellular target(s) of NC043 and explore the mechanism behind the inhibitory activity of NC043 against Wnt signaling, in this work, we generated biotinylated NC043 and captured CARF through mass spectrometry analysis. We found that NC043 binds to Cys-516 of CARF covalently. Further studies indicated that CARF interacts with Dvl in the nucleus, by which CARF potentiates the Dvl–c-Jun–β-catenin–TCF4 transcriptional complex and thus promotes transcription of Wnt target genes. Binding of NC043 to CARF disrupts CARF–Dvl interaction, thereby leading to inhibition of the Wnt signaling. The positive regulatory role of CARF in Wnt/β-catenin signaling is further confirmed by phenotypic analysis of CARF knockdown in zebrafish.

## Results

### CARF is the cellular target of NC043

**Figure 1:**
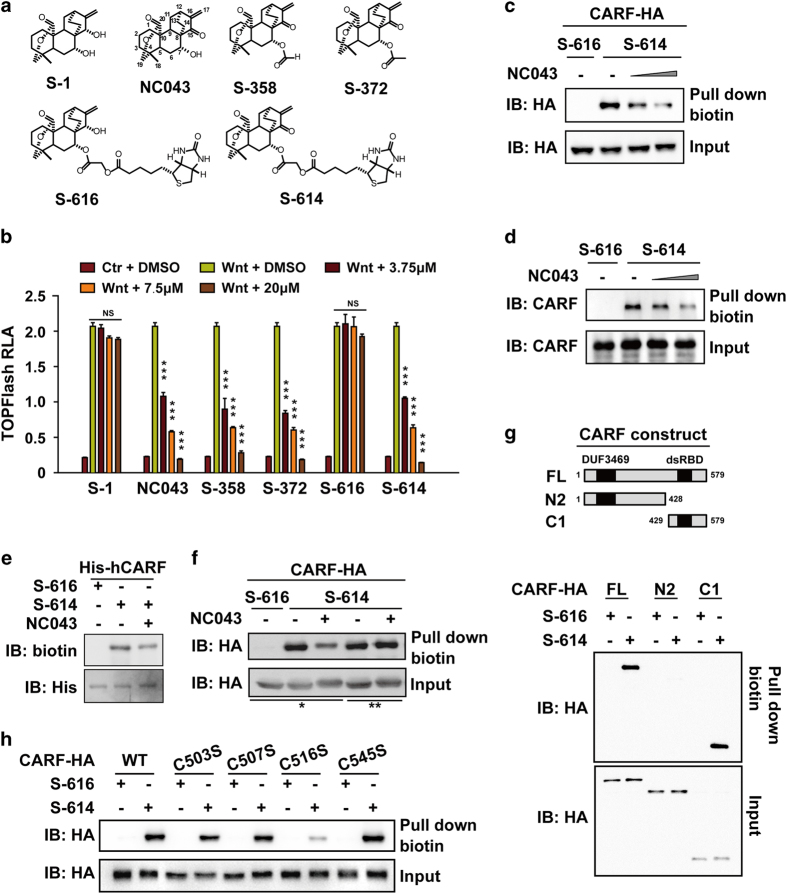
CARF is a target of NC043. (**a**) Chemical structures of NC043 and its analogs. (**b**) Effects of NC043 and its analogs on TOPFlash reporter activity. After 18 h of transfection, HEK293 cells were treated with the control (Ctr) conditioned medium (CM) or the Wnt3a CM plus the indicated small molecules for an additional 6 h before the luciferase activity assays. RLA, relative luciferase activity. Error bars indicate the s.d. of triplicate assays in one experiment. Each experiment was repeated at least three times. (*P-value<0.05; **P-value<0.01; ***P-value<0.001). (**c**) S-614 binds to CARF overexpressed in HEK293T cells. HEK293T overexpressed HA-tagged CARF were treated with 7.5 μm S-616 or S-614 plus with 0, 3.75 and 15 μm of NC043 for 1 h. The cell lysates were used for biotin-pulldown assay. The levels of CARF before and after pulldown were detected through western blotting analysis with HA antibody. (**d**) S-614 binds to endogenous CARF. After 1 h of 7.5 μm S-616 or S-614 plus with 0, 3.75 and 15 μm NC043 treatment, HEK293 cells were lysed, and followed by biotin-pulldown and western blotting analysis using a CARF specific antibody (anti-CARF). S-616 was used as a negative control. Error bars indicate the s.d. of triplicate assays in one experiment. Each experiment was repeated at least three times. (**e**) S-614 directly binds to purified CARF expressed in bacteria. The lysate of His6-tagged hCARF expressed *E.coli*. was incubated with Ni-beads for 1 h. After three times of wash, the beads were resuspended and incubated with small compounds (7.5 μm S-614 or S-616 and 15 μm NC043) as indicated for 1 h at 4 °C. Samples were subjected to biotin and His detaction. (**f**) NC043 binds to CARF covalently. After transfection, HEK293T cells were treated with 7.5 μm S-614 or S-616 and 15 μm NC043 as indicated, followed by biotin-pulldown and western blotting analysis. ‘*’ Cells were incubated in DMSO-contained medium for half-an-hour, and then treated with S-616/DMSO, S-614/DMSO or S-614/NC043 (lane 1-3) for 1 h; ‘**’ After S-614 pre-treatment, cells were incubated in S-614/DMSO or S-614/NC043 (lane 4–5) supplemented medium for additional 1 h. (**g**) Mapping the region on CARF responsible for binding NC043. Upper panel: schematic representation of full-length CARF (FL) and its truncations. After plasmids transfection and compounds incubation, HEK293T cells were used for biotin-pulldown and western blotting analysis. (**h**) C516 is the critical site for CARF to bind NC043. HEK293T cells expressed CARF (WT) and the indicated mutants were used for S614 binding assay followed by western blotting analysis.

To identify the cellular target (or targets) of NC043, we attempted to generate biotinylated NC043 without compromising its inhibitory activity for Wnt signaling. To figure out the modifiable sites on NC043, a series of its derivatives were designed with modifications on distinct chemical sites of NC043 (Figure 1a; Supplementary Figure S1). We then compared their inhibitory activities with that of NC043 using cell-based Wnt reporter assay. Our results showed that the analogs with modification on the 7-OH position such as S-358 and S-372 retained the Wnt-inhibitory activity; whereas S-1, with the C15 ‘=O’ replaced by ‘–OH’, lost completely the activity of inhibiting Wnt signaling (Figure 1b). Therefore, we attached biotin to the 7-OH position of NC043 and designated this analog as S-614, which has a molecular weight of 614 Da. We also generated a biotin-attached S-1 and designated it as S-616, which severed as a negative control (Figure 1a; Supplementary Figure S1). We verified their respective activity against Wnt signaling using reporter analysis. As expected, S-614 exhibited comparable activity with that of NC043, whereas S-616 showed no activity against Wnt signaling (Figure 1b).

Next, we treated SW480 cells with 15 μm S-614 or S-616 for 1 h, and subjected both whole-cell lysates and nuclear extracts of them to biotin-pulldown assay followed by mass spectrum (MS)-based label-free quantitative proteomic analysis. Overall, 91 candidate proteins from the whole-cell lysate samples and 75 ones from the nuclear extract samples showed significant quantification difference between S-614- and S-616-treated cells. Among these candidates, 8 proteins were found in both whole-cell lysate and the nuclear extract samples (Supplementary data set 1). We then knockdowned 6 of these 8 candidates (excluding 2 subunits of the condensin complex) in SW480 cells using the method of RNAi and examined their respective effects on the expression of *lef1*, a Wnt target gene, through the method of quantitative Real-time PCR. Our results showed that among the 6 candidates, only CARF knockdown exhibited an apparent effect on the expression levels of *lef1*, indicating the possibility of CARF as the cellular target of NC043 in Wnt signaling (Supplementary Figure S2). Next, we found that NC043 could effectively compete with S-614 for binding to overexpressed CARF in a dose-dependent manner (Figure 1c), and further detected an apparent interaction between the endogenous CARF and S-614 (Figure 1d). Moreover, in vitro binding assay using purified CARF showed that S-614 directly binds to CARF, which could be competed off by NC043 (Figure 1e). Altogether, these results suggested that CARF is the cellular target of NC043.

### NC043 covalently binds to CARF

NC043 has one Michael receptor, a group susceptible to react with electron donors, such as the –SH group in cysteine residue. As shown above, when this Michael receptor is modified as in the case of the analog S-1, the inhibitory activity against Wnt signaling is completely lost, indicating that the Michael receptor of NC043 has an essential role in its binding to CARF. One possibility is that this Michael receptor of NC043 forms a covalent bond with some cysteine residue of CARF. To test this possibility, we performed the competitive binding assay by adding S-614 and NC043 in sequence. If a covalent binding occurs between S-614 and CARF, S-614 would not be competed off by the subsequent treatment of NC043 due to the irreversible covalent bond; otherwise, a competitive effect between S-614 and NC043 would be observed regardless of the sequence of their treatment. Our results showed that when NC043 and S-614 were added to the cells simultaneously, NC043 could effectively compete with S-614 for binding to CARF (Figure 1f , lane 2 and 3). However, if the cells were pretreated with S-614 for half-an-hour, the subsequent treatment of NC043 would lose its ability to compete with S-614 for binding to CARF (Figure 1f, lane 4 and 5). These results suggest that S-614 binds to CARF covalently probably through targeting some cysteine residue of CARF via Michael reaction.

Next, we mapped the interaction region on CARF for NC043. Our results showed that NC043 binds to a short C-terminal fragment of CARF (designated as C1) (Figure 1g). To identify the potential cysteine residue critical in mediating the covalent binding between CARF and NC043, we generated single mutant for each of the four cysteine residues within this C1 fragment and compared their affinity for NC043 with that of the wildtype CARF (CARF^WT^). As shown in Figure 1h, only the mutation at the Cys-516 but not at the other sites of CARF, led to a significant decrease of binding with NC043. This suggests Cys-516 as the key site of CARF for binding NC043.

### CARF positively regulates Wnt/β-catenin signaling

**Figure 2:**
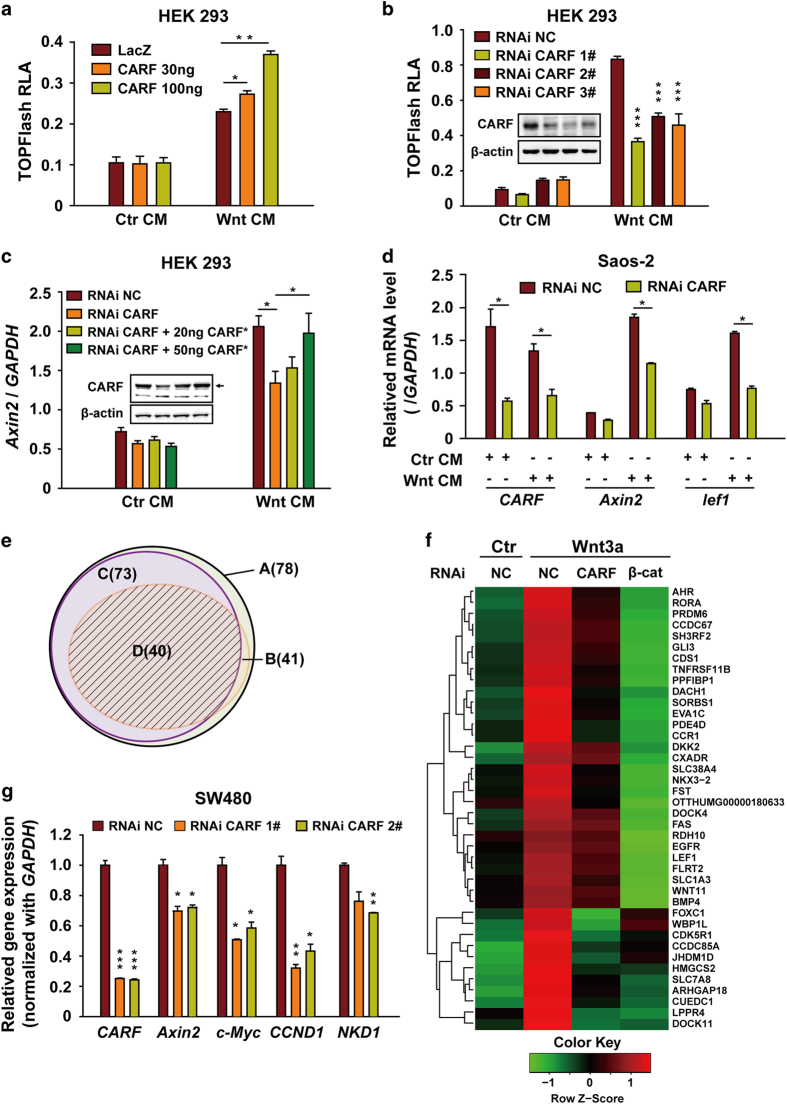
CARF promotes canonical Wnt signaling downstream of β-catenin accumulation**.** (**a**) Overexpressed CARF promotes Wnt signaling. After transfection as indicated, HEK293 cells were used for TOPFlash reporter assay. (**b**) Knockdown of CARF represses Wnt3a-induced TOPFlash activity. After CARF knockdown with the indicated siRNAs for 48 h, HEK293 cells were subjected to reporter assay. (**c**) Expression of *Axin2* was decreased by CARF knockdown and restored by CARF overexpression. Inner panel shows the expression levels of CARF with β-actin as the loading control. ‘CARF*’ indicates that the plasmid is a siRNA-resistant one. (**d**) Expression of Wnt target genes was inhibited by CARF knockdown in Saos-2 cells. After 42 h of CARF RNAi and additional 6 h of Wnt CM treatment, cells were subjected to RT-PCR. (**e**, **f**) Profile of genes regulated by Wnt, β-catenin and CARF. (**e**) Venn diagram for the relationship between CARF and β-catenin regulated genes among Wnt-responded ones. A, Set of genes responding to Wnt3a; B, Set of genes with a decreased expression after CARF knockdown; C, Set of genes downregulated by β-catenin knockdown; D, The intersection of B&C. (**f**) Expression pattern of the 40 genes from the set D described in **e**. All the analyses were based on two independent experiments. (**g**) Knockdown of CARF repressed expression of the Wnt target genes in SW480. After 48 h of CARF RNAi, cells were subjected to RT-PCR. Error bars indicate the s.d. of triplicate assays in one experiment. Each experiment was repeated at least three times.

As the cellular target of NC043, a Wnt pathway inhibitor, CARF might have some role in Wnt signaling pathway. To investigate the possible function of CARF in Wnt signaling, we first examined the effects of CARF on TOPflash luciferase activity. We found that the Wnt3a-induced TOPflash reporter activity could be further enhanced by CARF overexpression in a dose-dependent manner, whereas the basal Wnt signaling activity is not affected by CARF overexpression (Figure 2a). Consistent with this, knockdown of CARF led to decreased TOPflash reporter activity (Figure 2b). We next assessed the effects of CARF on the expression of Wnt target gene *Axin2*. Consistent with the results obtained from the reporter assay, CARF knockdown repressed Wnt-promoting *Axin2* expression and this effect could be rescued by transfection of siRNA-resistant CARF plasmid (CARF*, Figure 2c). These results indicated that CARF positively regulates Wnt signaling. As mentioned previously, CARF was reported to regulate p53 pathway [15, 16], and p53 pathway could regulate canonical Wnt signaling through transactivating microRNA-34 [21]. To examine whether the upregulating effect of CARF on Wnt signaling is related to its function in p53 pathway, we carried out CARF knockdown experiment in Saos-2 cell line—a p53-null *Osteosarcoma* cell line. Q-PCR results showed that knockdown of CARF dramatically decreased expression of Wnt target genes induced by Wnt3a in Saos-2 cells (Figure 2d), indicating that the function of CARF in Wnt signaling is independent of its activity in p53 pathway.

To examine quantitative changes in Wnt target gene expression regulated by CARF, we analyzed by microarrays the gene expression profiles of Saos-2 cells after CARF deprivation. β-catenin siRNA was used as a positive control. According to our result, 78 genes were up-regulated by Wnt3a stimulation with at least 1.5-fold change compared with the control group, including the well-known Wnt target genes, such as *Axin2, lef1* and *EGFR*. To determine the effects on Wnt signaling, we defined a threshold for valid inhibition as 20% decrease in target genes expression on the treatment of CARF or β-catenin knockdown. Among the Wnt3a-induced genes, 73 were decreased by β-catenin knockdown and 41 were decreased by CARF knockdown, with 40 genes overlapped between them (Figure 2e and f **;** Supplementary Figure S3A and B). We picked several genes randomly from these 40 genes listed in Supplementary data set 2 and verified the change of their expression via qPCR (Supplementary Figure S3C). These data confirmed that CARF positively regulates Wnt signaling and also indicated that CARF most likely serves as a regulator instead of a key factor as β-catenin in the Wnt pathway.

### CARF interacts with Dvl directly

Our previous work showed that NC043 inhibits Wnt signaling at the level of TCF/LEF1 transcriptional complex, indicating that CARF may function in the nucleus. As expected, neither LRP6 phosphorylation nor β-catenin phosphorylation was affected by CARF knockdown (Supplementary Figure S4A and B). We then assessed the expression of Wnt target genes after CARF knockdown in SW480 cell line, which harbors a loss-of-function mutation in *APC* gene. APC is a critical component of the β-catenin destruction complex. Loss of the functioning APC would result in β-catenin accumulation, subsequent nuclear translocation and consequently overactivation of Wnt pathway. As shown in Figure 2g, knockdown of CARF in SW480 cells decreased the expression of the Wnt target genes, indicating that CARF functions downstream of the β-catenin destruction complex. To exclude that CARF functions at the level of β-catenin accumulation, we examined the effects of CARF on TOPflash reporter activity in cells treated with LiCl or transfected with ΔN β-catenin. LiCl (lithium chloride) is a GSK3 inhibitor, which prevents the function of β-catenin degradation complex. ΔN β-catenin is an N-terminal 1-45aa β-catenin truncation resistant to the regulation of the degradation complex. Hence, both LiCl treatment and ΔN β-catenin transfection lead to the accumulation of β-catenin. As shown in Supplementary Figure S4C, overexpression of CARF further enhanced the TOPFlash activity induced by LiCl or transfection of ΔN β-catenin in HEK293 cells, indicating that CARF functions downstream of β-catenin accumulation. Moreover, we noticed that nuclear-cytoplasmic shuttling of β-catenin induced by Wnt3a was also not affected by CARF RNAi (Supplementary Figure S4D). Together, these results suggested that CARF may function at the level of TCF/LEF1 transcriptional complex in the nucleus.

**Figure 3:**
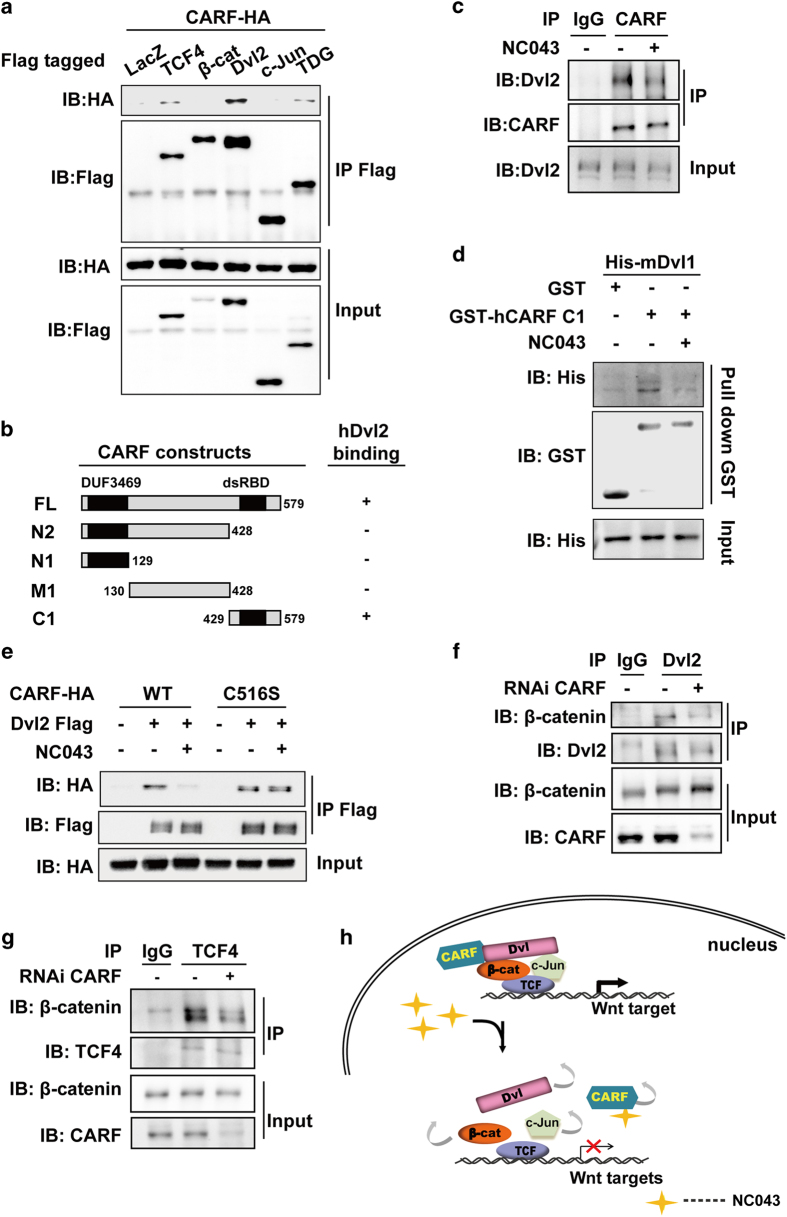
CARF facilitates assemble of the multi-components transcriptional complex through interacting with Dvl**.** (**a**) CARF interacts with Dvl2. HEK293T cells were transfected as indicated, and followed by immunoprecipitation (IP) using anti-Flag and western blotting analysis. (**b**) Schematic representation of the full-length and truncated CARF with the indicated binding affinity for Dvl2. (**c**) CARF interacts with Dvl2 *in vivo* and this interaction is disrupted by NC043. Nuclear extract of HEK293 cells with or without NC043 treatment were subjected to IP using anti-CARF followed by immunoblotting. IgG was used as a negative control for the IP analysis. (**d**) NC043 disrupts CARF-Dvl2 interaction *in vitro.* GST-hCARF and His-mDvl1 expressed in *E.coli* were used for GST-pulldown with or without NC043 incubation, followed by western blotting analysis. (**e**) NC043 disrupts the interaction of Dvl2 with WT CARF but not with C516S mutant. HEK293T cells transfected with indicated plasmids were subjected to IP analysis using anti-Flag followed by western blotting analysis using anti-HA. (**f**, **g**) CARF knockdown decreases Dvl–β-catenin association (**f**) and TCF4–β-catenin association (**g**). The nuclear extracts from CARF-deprived HEK293T cells were subjected to IP using anti-Dvl2 and anti-TCF4 followed by western blotting analysis. (**h**) A model for how CARF facilitates TCF transcriptional activity and NC043 inhibits Wnt signaling. On Wnt stimulation, CARF facilitates assembly of the Dvl–c-Jun–β-catenin–TCF complex through interacting directly with Dvl. When NC043 is present, NC043 binds to CARF covalently via targeting its C516 residue, which disrupts CARF–Dvl interaction and consequently impairs the TCF multi-components complex.

To explore how CARF may function at the level of TCF transcriptional complex, we first examined the interaction between CARF and various components of TCF/LEF1 transcriptional complex, including TCF4, β-catenin, Dvl(s), c-Jun and TDG [22]. Among these proteins, Dvl showed the strongest binding with CARF (Figure 3a). To verify the interaction between CARF and Dvl, we isolated the nuclear fraction of HEK293 cells and performed endogenous binding assay. As shown in Supplementary Figure S5A, we detected an apparent interaction between endogenous CARF and Dvl2. CARF also interacted with Dvl1 and Dvl3, the other two members of human Dvl family (Supplementary Figure S5B).

Next, we mapped the Dvl-interacting region on CARF, which showed that the truncation C1 is sufficient for CARF to interact with Dvl (Figure 3b; Supplementary Figure S5C). As C1 is also the region responsible for binding NC043 as we revealed above, we then asked whether binding of NC043 to CARF affects the interaction of CARF with Dvl. As shown in Figure 3c, NC043 treatment led to a dramatic decrease of the interaction between the endogenous CARF and Dvl, suggesting that NC043 could disrupt CARF–Dvl interaction. Our further GST-pulldown assay using recombinant CARF and Dvl indicated that the interaction between CARF and Dvl is a direct one, which could be disrupted by NC043 (Figure 3d). However, NC043 could not affect the interaction of CARF^C516S^ with Dvl (Figure 3e), suggesting that NC043 disrupts CARF–Dvl interaction through targeting Cys-516 of CARF. Taken together, these results suggested that CARF interacts directly with Dvl, and NC043 could disrupt their interaction by targeting the Cys-516 residue of CARF.

### CARF potentiates β-catenin/TCF4 transcriptional complex

Previously, we reported that Dvl scaffolds the complex of c-Jun–β-catenin–TCF4 to facilitate TCF4 transcriptional activity [11]. We then asked whether CARF–Dvl interaction may affect the assembly of the Dvl–c-Jun–β-catenin–TCF4 transcriptional complex. As shown in Figure 3f, CARF knockdown led to a reduced association between β-catenin and Dvl2, without affecting Dvl2 or β-catenin subcellular distribution (Supplementary Figure S4D). Similar effects of CARF on β-catenin–TCF4 interaction was observed (Figure 3g). Consistent with this, CARF overexpression promoted interaction of β-catenin with TCF4. Of note, both CARF^WT^ and CARF^C516S^ could enhance the association between TCF4 and β-catenin by the same level; however, the treatment of NC043 abolished the effects of CARF^WT^, but not that of CARF^C516S^, on the β-catenin–TCF4 interaction (Supplementary Figure S6A). These results suggested that CARF promotes the assembly of the multi-component TCF4 transcriptional complex, and this function of CARF does not require the Cys-516 residue. To further confirm the important role of CARF in mediating the assembly of the TCF4 transcriptional complex, we performed the chromatin immune co-precipitation (ChIP) assay. As shown in Supplementary Figure S6B and C, both β-catenin and Dvl2 were enriched on the TCF-binding site (TBE) (also known as Wnt response element [WRE]), of Wnt target genes *Axin2* and *NKD1* in SW480 cells. However, this enrichment of β-catenin and Dvl2 on TBE was dampened by the knockdown of CARF. By contrast, the association of TCF4 on TBE was not affected by CARF deprivation. Next, to investigate the effect of Wnt stimulation on interactions of CARF with the components in TCF4 transcriptional complex, we performed endogenous Co-IP assay. As shown in Supplementary Figure S6D, CARF interacted with Dvl in the absence of Wnt stimulation. Upon Wnt stimulation, the interaction between CARF and Dvl slightly increased probably due to a slight increase of Dvl nuclear accumulation; while the interaction between β-catenin and CARF showed an apparent increase (Supplementary Figure S6D). On the basis of these results and our previous findings [11], we tend to believe that CARF constantly interacts with Dvl, and on Wnt stimulation, the CARF–Dvl complex may move to the TCF4 transcriptional complex, where CARF facilitates TCF4 transcriptional activity by further strengthening the multi-components complex (Figure 3h). Binding of NC043 to CARF disrupts CARF–Dvl interaction, hence destabilizing the TCF4 multi-components complex and consequently leading to inhibition of Wnt signaling (Figure 3h).

### CARF facilitates Wnt signal transduction in zebrafish embryogenesis

**Figure 4:**
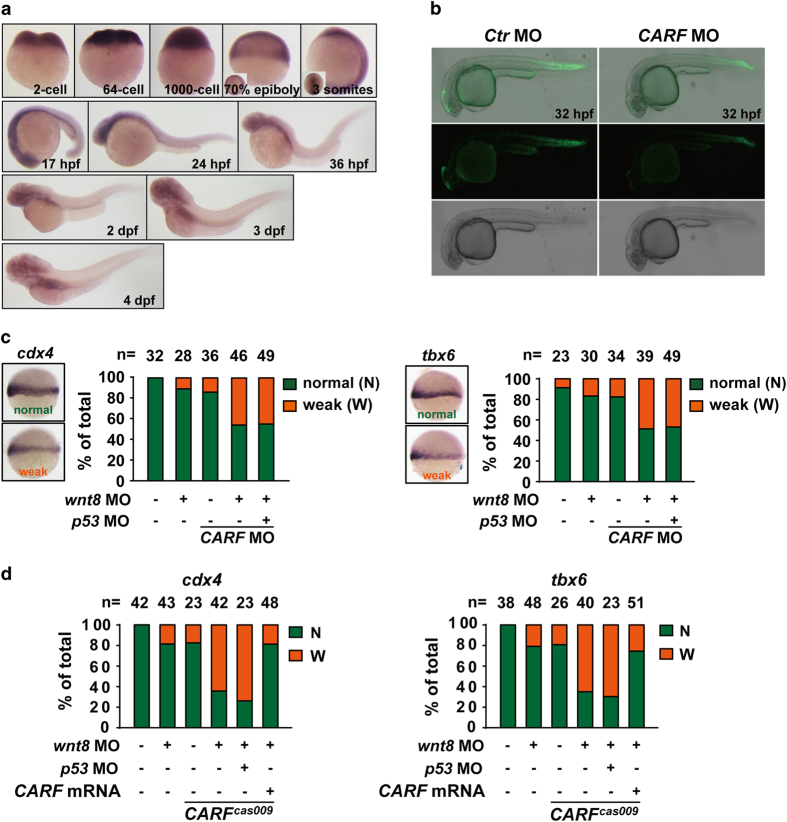
CARF facilitates Wnt/β-catenin signaling in zebrafish embryogenesis. (**a**) The whole mount *in situ* analysis of CARF expression in zebrafish embryogenesis. The embryos at indicated developmental stages were fixed for *in situ* hybridization with *CARF* probe. (**b**) CARF knockdown attenuates Wnt activation in Tg(*tcf:egfp*) line. (**c**, **d**) *CARF* ^*cas009*^ mutant or CARF morphants show reduced Wnt signaling activity via a p53-independet manner. Embryos injected with the indicated morpholinos or morpholino-mRNA mixture were fixed at the 60% epiboly stage and then analyzed by WISH with *cdx4* (left) or *tbx6* (right) probe. The relative expression of each marker was classified into two categories: normal (N) or weak (W). The number of the total embryos scored (*n*) is shown on the top of each bar.

To understand the biological function of CARF, we analyzed *CARF* expression in zebrafish embryos. The *in situ* hybridization analysis shows a maternal and then ubiquitous expression pattern of *CARF* in different developmental stages up to 4day-post fertilization (dpf) (Figure 4a). To evaluate the regulatory role of CARF in Wnt signaling, we microinjected CARF targeting morpholino at 1-cell stage of Wnt reporter Tg(*tcf:egfp*) zebrafish embryos, which displays reduced EGFP signaling compared with that of control morpholino-injected embryos (Figure 4b). During zebrafish embryogenesis, canonical Wnt/β-catenin signaling is critical for mesoderm induction [23]. Suppression of Wnt signaling via *wnt8* morpholino injection is known to cause mesodermal defects and decreased expression of *cdx4* and *tbx6*, two known ventrolateral mesodermal marker downstream of canonical Wnt signaling [24, 25]. We found CARF knockdown via morpholino injection enhance such defects (Figure 4c), which could not be rescued by p53 knockdown. Moreover, we recaptured this defective phenotype in *CARF* ^*cas009*^ mutants (Figure 4d). These results further indicate CARF is a positive regulator in Wnt signal transduction.

### CARF is essential for hematopoietic stem/progenitor cell formation and fin regeneration in zebrafish

**Figure 5:**
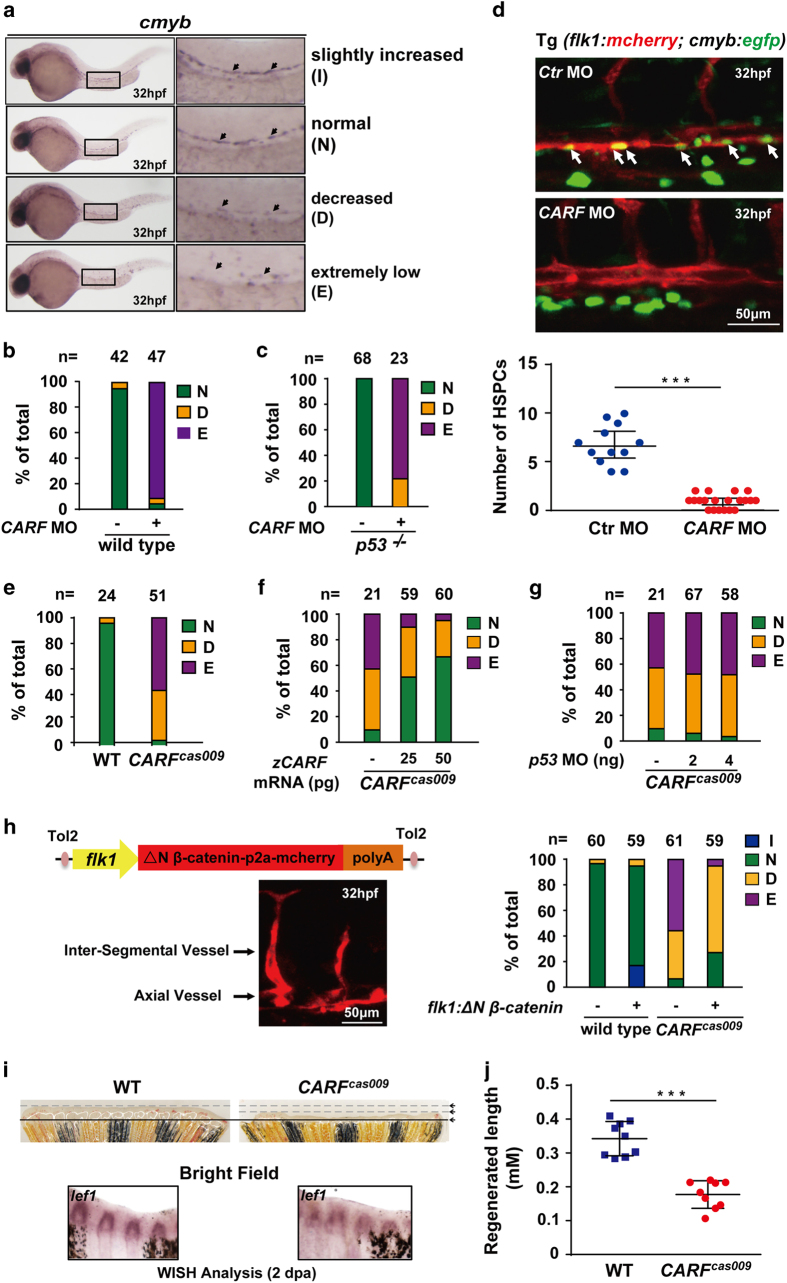
Loss of CARF attenuates HSPC formation and caudal fin regeneration**.** (**a–d**) Knockdown CARF limits HSPC formation. Zebrafish embryos at 32 hpf were fixed for *in situ* hybridization with *cmyb* probe, and then classified into three categories: (**a**) Representative image for slightly increased (I), normal (N), decreased (D) or extremely low (E) level of *c-myb* WISH analysis. Knockdown CARF dramatically inhibits HSPC formation (**b**) which could not be rescued via p53 MO co-injection (**c**) and further validated by the live image of HSPC budding events in AGM of zebrafish Tg(*flk1:mcherry;cmyb:egfp*) line (**d**). (**e–g**) *CARF* ^*cas009*^ mutant exhibits reduced HSPC formation via a p53-independent manner. Injection of zebrafish CARF (zCARF) mRNA (**f**) but not p53 MO (**g**) restores decreased *cmyb* expression in *CARF* ^*cas009*^ mutants (**e**). (**h**) Schematic diagram of Tol2 transposase-mediated transient transgenesis of endothelial-specific promoter (*flk1*)-derived expression of constitutive activated β-catenin (ΔN β-catenin) and mCherry chimera protein in zebrafish embryos, which rescues hematopoietic defects in *CARF* ^*cas009*^ mutants while slightly increases HSC formation in wildtype zebrafish. (**i**, **j**) Delayed fin regeneration of *CARF* ^*cas009*^ mutants. Adult *CARF* ^*cas009*^ zebrafish were executed caudal fin amputation and then cultured for regeneration. The regenerated sections were cut down at 2 dpa for either WISH analysis with *lef1* probes or length measurement.

Wnt signaling has a key and conserved role in hematopoietic stem/progenitor cell (HSPC) generation [26]. Here, we examined whether weakening Wnt signaling by CARF deprivation affects the determination of HSPC by examining the expression of *cmyb* at 32 h post-fertilization (hpf), which was classified as standards shown in Figure 5a. Injection of CARF morpholino severely reduced the number of *cmyb* positive cells (Figure 5b and d), which could not be rescued in *p53* mutants (Figure 5c). This phenotype was recaptured in *CARF* ^*cas009*^ mutants compared with that of wildtype embryos (Figure 5e), which could be partially rescued by ectopic expression of wildtype CARF (Figure 5f), but not be rescued by p53 knockdown (Figure 5g). To further investigate whether CARF knockdown or *CARF* ^*cas009*^ mutation-caused defective HSPC induction results from attenuated Wnt signaling, we carried out transient transgenesis to induce endothelial-specific expression of constitutive activated zβ-catenin2 (N-terminal 1-47aa truncation of zβ-catenin2 resistant to β-catenin degradation complex). Confocal live imaging shows a vascular-specific expression pattern after transgenesis, and *c-myb* WISH analysis displays a rescued definitive hematopoiesis (Figure 5h). Moreover, although *CARF* ^*cas009*^ mutants carry defective definitive hematopoiesis, about 17% mutants survived into adulthood at 6 month post-fertilization. We tested the role of CARF in caudal fin regeneration, which is a known Wnt/β-catenin signaling-dependent process [5, 27, 28]. As expected, compared with wildtype fish, *CARF* ^*cas009*^ homozygotes have dramatic shorter regenerated caudal fin 48 h after resection (Figure 5i and j), and the expression of Wnt target gene *lef1* (Figure 5i) is significantly reduced. These data suggest that CARF regulates zebrafish caudal fin regeneration through facilitating Wnt signaling.

## Discussion

Previously, we identified the small-molecule compound NC043 as an inhibitor of Wnt/β-catenin signaling. In this study, we generated biotinylated NC043 (S-614) to identify CARF as the cellular target for NC043 and uncovered the mechanism behind the inhibitory activity of NC043 against Wnt/β-catenin signaling.

NC043 contains a ‘Michael acceptor’ which is susceptible to receive electron from electron donors, such as –SH in cysteine. Our results showed that this Michael acceptor is critical for NC043 to bind to CARF. Further analysis indicated that this ‘Michael acceptor’ of NC043 forms a covalent bond with the Cys-516 residue of CARF. Next, we uncovered that CARF is a positive regulator of Wnt signaling by interacting with Dvl. Dvl is a key component of Wnt/β-catenin signaling and its activation results in the formation of LRP5/6 signaling complex (also known LRP5/6 signalosome), allowing for accumulation of β-catenin and consequently activation of the downstream TCF transcriptional complex. In addition to functioning in the cytoplasm, Dvl also has a critical role in the nucleus. We previously reported a significant role of Dvl in the nucleus by forming a complex with c-Jun, β-catenin and TCF4 to facilitate the transcriptional activation of the Wnt target genes [11]. Here, we found that knockdown of CARF leads to reduced recruitment of both β-catenin and Dvl to the TBE region, suggesting an important role of CARF in the assembly of the Dvl–c-Jun–β-catenin–TCF4 complex.

Of note, both NC043 and Dvl target the C-terminal C1 fragment of CARF. Our results suggested that the Cys-516 residue of CARF is critical for binding NC043 but seems dispensable for its interaction with Dvl. As such, NC043 could disrupt the interaction of CARF^WT^ with Dvl, but not that of CARF^C516S^ with Dvl. It remains unknown whether binding of NC043 disrupts the CARF–Dvl complex interface directly or induces a conformational change of CARF, leading to its dissociation from Dvl. Three-dimensional structural study of CARF-NC043 and CARF–Dvl is expected to provide valuable insights into the molecular details behind the assembly of these two complexes.

Wnt/β-catenin signaling has essential roles in early embryogenesis and organ/tissue formation, including HSPC determination [29]. In this study, we generate CARF morpholino and *CARF* ^*cas009*^ mutants, both of which show reduced Wnt signal activity, followed with dramatically inhibited HSPC marker expression and endothelial-to-hematopoietic transition events. Although about 17% *CARF* ^*cas009*^ mutant zebrafish survive into adulthood at 6 months post-fertilization, they display reduced regeneration capability after caudal fin amputation with decreased Wnt target gene: *lef1* expression. These results are also consistent with the conclusion that CARF serves as an important positive modulator, but not major transducer in Wnt/β-catenin signaling.

CARF was previously reported to function in p53 pathway. Several studies have indicated the involvement of CARF in regulating tumor cell growth. Interestingly, CARF seems to exert both suppressing and promoting effects on tumor growth, probably depending on its distinct expression levels in these tumor cells [29–31]. Here, we uncovered a positive regulatory role of CARF in Wnt/β-catenin signaling by interacting with Dvl. Of note, during the preparation of this manuscript, Fan *et al*. [32] revealed that CARF activates Wnt signaling though competing with ICAT for β-catenin. In our work, we didn't observe an apparent interaction of β-catenin with CARF in the absence of Wnt stimulation; while our results indicated that CARF may interact with Dvl independent of Wnt stimulation. Wnt pathway controls many aspects of cell behaviors and its deregulation is closely related with various human diseases including many cancers. Although great efforts have been made to develop compounds targeting Wnt pathway, drugs developed for this pathway are far from satisfactory due to limited drug targets identified in Wnt pathway. Our findings here revealed CARF as a penitential new therapeutic target for Wnt-driven diseases. Aberrantly unregulated CARF activity may contribute to overactivation of Wnt signaling and hence to the growth of tumor cells. These tumor cells are very likely to be sensitive to NC043 treatment. Thus, screening by a combination of NC043 treatment and CARF knockdown against various tumor cell lines may lead to identification of the specific tumor types that would be sensitive to CARF-targeted personalized cancer therapy. Further studies along this line will be carried out to explore the potential of CARF as a new drug target.

## Materials and Methods

### Chemical synthesis

The syntheses of NC043 and S-1 were described as before [3] and that of S-358, S-372, S-616 and S-614 are described in the Supplementary Information **.** HEK293, HEK293T, Saos-2 and SW480 cells were transfected with plasmids using Lipofectamine 3000 Transfection Reagent (Invitrogen, Carlsbad, CA, USA), or siRNA using Lipofectamine RNAiMAX (Invitrogen) according to the manufacturer’s instructions. For reporter gene assays, HEK293 cells were seeded in 24-well plates. Each well of HEK293 cells was transfected with 250 ng of plasmids in total, including 20 ng of TOPFlash, 25 ng of EGFP-C1 and other plasmid(s) as indicated. The LacZ plasmid was added to equalize the total amount of transfected DNA to 250 ng. In Figure 3b, HEK293 cells were transfected with 20 nm of indicated siRNA 24 h before plasmids transfection. In Supplementary Figure S3C, 2.5 ng ΔN-β-catenin was transfected. Eighteen hour post-transfection, cells were treated with Wnt3a conditioned medium (20 mm LiCl in Supplementary Figure S3C) or control medium for an additional 6 h and then lysed for luciferase assays. The GFP expression levels were determined for normalization as described previously [33].

### Cytosol and nucleus fractionation

Cells were grown in 60 mm dishes, harvested with a cell scraper into 1.5 ml of PBS and spun at 700×*g* for 10 min. The pelleted cells were resuspended in buffer A (10 mm HEPES, pH 7.9, 1.5 mm MgCl_2_, 10 mm KCl, 0.5 mm DTT, 10 mm NaF, 2 mm Na_3_VO_4_, 1 mm pyrophosphoric acid and Complete protease inhibitors (Roche, Mannheim, Germany)) and incubated on ice for 10 min. Cells were then passed through a 0.4-mm needlepoint to break down the cell membrane and were then centrifuged at 700×*g* at 4 °C for 10 min. The supernatant was collected and centrifuged at 100,000×*g* at 4 °C for 1 h. The supernatant from the ultracentrifugation was collected as the cytosolic fraction. For nuclear protein extraction, the pellet from the 700×*g* centrifugation was washed by buffer A, resuspended in buffer C (20 mm HEPES, pH 7.9, 1.5 mm MgCl_2_, 420 mm NaCl, 0.2 mm EDTA, 10 mm NaF, 2 mm Na_3_VO_4_, 1 mm pyrophosphoric acid and Complete protease inhibitors) and incubated on ice for 30 min. Nuclear extracts were recovered from the supernatants after centrifugation at 100,000×*g* at 4 °C for 1 h.

### Biotin-pulldown assay

1 h post-treatment of biotinylated small molecule as described, cells were lysed with lysis buffer (50 mm Tris-HCl, 150 mm NaCl, 1% (v/v) Triton X-100, 5 mm EDTA, pH 7.4) containing proteinase inhibitors and centrifuged at 16,000×*g* for 15 min at 4 °C. Overall, 20 μl supernatant was then used as the input by adding 20 μl 2×SDS loading buffer, and the remains were mixed with Streptavidin Agarose (Novex, Carlsbad, CA, USA). After 1 h incubation, the beads were washed three times and resuspended in 40 μl 2×SDS loading buffer.

### Quantitative real-time PCR with reverse transcription

The total RNA was isolated with a TRIzol kit (Invitrogen). Reverse transcription was performed using SuperScript III First-Strand Synthesis System for RT-PCR (Invitrogen) according to the manufacturer's instructions. Quantitative RT-PCR was performed with the SYBR Premix Ex Taq (Takara, Kusatsu, Japan) on the ABI PRISM 7500 system (Applied Biosystems, Waltham, MA, USA). The specific primers used for detecting genes are listed in Supplementary Table S3.

### Endogenous co-immunoprecipitation

Cells were lysed with lysis buffer (50 mm Tris-HCl, 150 mm NaCl, 1% (v/v) Triton X-100, 5 mm EDTA, pH 7.4) supplemented by protease inhibitors and centrifuged at 16,000×*g* for 15 min at 4 °C. Overall, 25 μl supernatant was then used as the input by adding 25 μl 2×SDS loading buffer, and the remains were incubated with the indicated antibodies overnight (or 4 h at least) at 4 °C followed by centrifugation. The supernatant collected was then mixed with Protein A/G PLUS-Agarose (Santa Cruz Biotechnology, Santa Cruz, CA, USA) for 2 h. IgG was used as a control. The beads were washed three times and resuspended in 40 μl 2×SDS loading buffer.

### GST-pulldown and in vitro binding assay

Recombinant proteins His6-tagged mDvl1, GST-hCARF and GST were expressed in *E. coli BL21(DE3).* For GST-pulldown assay, GST alone or GST-hCARF was purified from *E. coli* using Glutathione Sepharose 4 Fast Flow beads (GE Healthcare, Uppsala, Sweden), whereas His6-tagged mDvl1 was lysed using BugBuster Protein Extraction Reagent (Novagen, Billerica, MA, USA) without further purification. Purified GST or GST-hCARF was then mixed with His6-tagged mDvl1 plus DMSO or NC043 as indicated for 2 h. Overall, 20 μl mixture was collected as input by adding equal volume of 2×SDS loading buffer, and the remain was centrifuged. The beads were washed for 3 times and resuspended in 40 μl 2×SDS loading buffer.

### Microarray expression analysis

After siRNA transfection (control, CARF or β-catenin siRNA) for 42 h and additional 6 h of control or Wnt3a incubation, the total RNA was extracted from Saos-2 cells using TRIzol (Invitrogen). Two pairs of the following samples were prepared: si-control plus control CM (C-NC), si-control plus Wnt3a (W-NC), si-CARF plus Wnt3a (W-CARF) and si-β-catenin plus Wnt3a (W-β-cat). The aRNA amplification, purification, fragmentation and hybridization were peformed as described in the user manual of GeneAtlas 3' IVT Express Kit (Affymetrix, Santa Clara, CA, USA), and Affymetrix Human Genome U219 Array Strip was used. The differentially expressed gene probesets in W-NC vs C-NC, W-CARF vs W-NC and W-β-cat vs W-NC comparisons were selected in two biological replicates, using following thresholds respectively: (a) W-NC/C-NC>1.5; (b) W-CARF/W-NC<0.8; (c) W-β-cat/W-NC<0.8. Different probe values of same gene were integrated by calculating the sum to represent gene expression levels. In W-NC vs C-NC comparison, inhibitory proportions *I*_W-CARF_ by W-CARF and *I*_W-β-cat_ by W-β-cat were calculated as *I*_W-CARF (or W-β-cat)_=(*X*_W-NC_-*X*_W-CARF (or W-β-cat)_)/(*X*_W-NC_-*X*_*C-*NC_), where *X* denotes expression level. Genes whose upregulations were inhibited by W-CARF and W-β-cat were defined as: (a) W-NC/C-NC>1.5, (b) *I*_W-CARF_>20% and (c) *I*_W-β-cat_>20%. The heatmap showing the differentially expressed genes were generated using in-house scripts of R language. The color shows the normalized expression level of gene j, calculated as *Z*_i,j_=|*X*_i,j_-*μ*|/*μ*, where *X*_i,j_ denotes the mean expression level of gene *j* in samples of condition i, and μ denotes the mean of all samples.

### Microinjection and CRISPR/Cas9 mutagenesis

The mRNA was synthesized *in vitro* by SP6 mMessage mMachine Transcription Kit (Ambion, Austin, Texas, USA). The *CARF* gRNA was synthesized as previously described [34]. The information of the *CARF* gRNA target site was shown in Supplementary Table S5. The zebrafish optimized *cas9* mRNA was synthesized *in vitro* from the pCS2-nCas9n plasmid (Addgene, Cambridge, MA, USA, #47929) as previously described [35]. For the ectopic expression, Tol2 transposon-mediated transient transgenesis was applied and performed as previously described [36]. flk1-△N β-catenin-p2a-mcherry transgene constructs within Tol2 vectors (~45 ng/μl) were mixed with transposase mRNA (~50 ng/μl ) and 0.2 m KCl, and then injected into 1-cell stage embryos [37]. *wnt8* morpholinos (MOs, *wnt8*-ORF1 MO+*wnt8*-ORF2 MO) (0.6 ng, the sequences have been described previously [24]), *CARF* morpholino (MO) (5′-CCTCTTCTTGCCGCCATCACTCTAA-3′, 2 ng), and *p53* morpholino (MO) (5′-TCTTGGCTGTCGTTTTGCGCCATTG-3′, 4 ng) were injected into 1-cell stage embryos. All MOs were ordered from Gene Tools. For CRISPR/Cas9-mediated genome editing, *CARF* gRNA (100 pg) and *cas9* mRNA (400 pg) were co-injected into one-cell stage embryos. The putative mutation was confirmed by genomic DNA sequencing (PCR primers show in Supplementary Table S5).

### Whole mount *in situ *hybridization analysis

Probes were transcribed *in vitro *by T7 polymerase (Ambion) with Digoxigenin RNA Labeling Mix (Roche, Mannheim, Germany). One color WISH was performed as described previously [38]. Images were photographed by Olympus SZX16 microscope with Olympus DP80 CCD (OLYMPUS, Tokyo, Japan).

### Caudal fin amputations for regeneration

Zebrafish, 6-8 months of age, were used for caudal fin amputations. Fish were anaesthetized in tricaine and amputations were made by using a razor blade, removing half of the fin [27]. Animals were allowed to regenerate for 2 days in water kept at 31-33 °C; these temperatures facilitate more rapid regeneration than more commonly used temperatures of 25–28 °C [39]. Then, fish were anaesthetized, and the fin regenerate was removed for analyses. *lef1* probes were transcribed *in vitro *by T3 polymerase (Ambion) with Digoxigenin RNA Labeling Mix (Roche). *In situ *hybridization of zebrafish fins was performed as previously described [40].

### Live imaging analysis

Live transgenic zebrafish embryos were anesthetized in 0.03% Tricaine (Sigma-Aldrich, St. Louis, MO, USA) and mounted in 1% low-melt agarose. The confocal images were taken by ZEISS LSM 710 scanning confocal microscope (ZEISS, Oberkochen, Germany). *c-myb:egfp* & *flk1:mcherry* double positive cells were counted in projections of z-stack images. The Tg (*tcf: egfp*) transgenic embryos were anesthetized with 0.03% Tricaine (Sigma-Aldrich), mounted in 4% methylcellulose and imaged via Zeiss Axio Zoom V16 microscope equipped with a Zeiss Axio Cam MRm digital camera (ZEISS).

### Statistics analysis

Data were analyzed with the Graphpad Prism 5 software (GraphPad Software, La Jolla, CA, USA) using the two-tailed Student’s *t*-test. The plot error values were calculated by s.e.m. All data in this study were repeated for at least twice.

## Notes

Supplementary Information

Supplementary Data Set 1

Supplementary Data Set 2
