# Supplementary material for: Chemical biology reveals CARF as a positive regulator of canonical Wnt signaling by promoting TCF/β-catenin transcriptional activity
Source: Cell Discov. 2017 Jan 31;3:17003–. doi: 10.1038/celldisc.2017.3 (PMC5387711; doi:10.1038/celldisc.2017.3)
Supplement: Supplementary Information [file celldisc20173-s1.pdf]

SUPPLEMENTARY INFORMATION

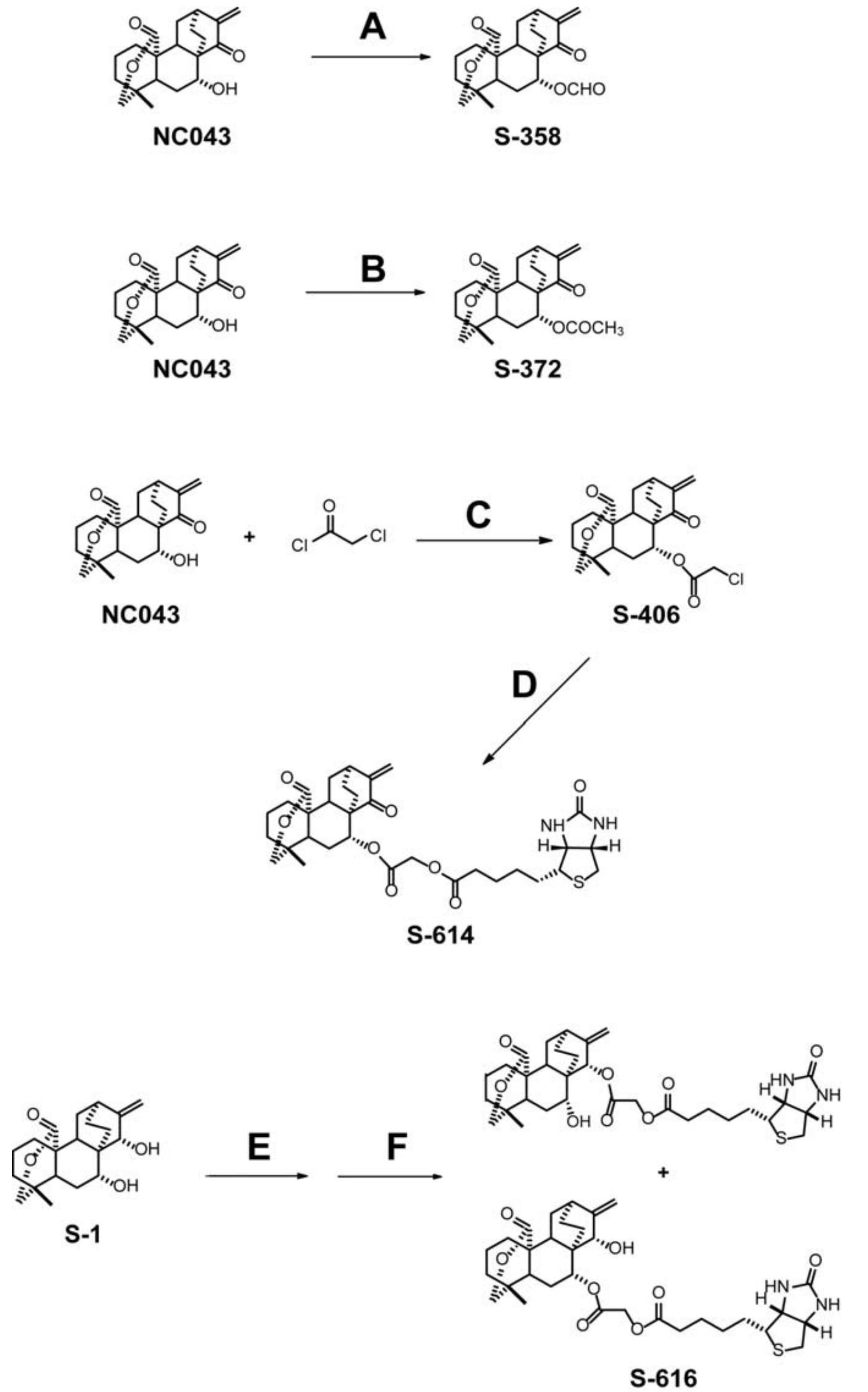

Supplementary Figure S1. Synthetic scheme for S-358, S-372, S-614 and S-616. **(A)** NC043 was injected into the prepared reaction system (($(COCl)_2$, DCM and DMF), stirred for 2 h at room temperature. Yield: 94%. **(B)** NC043, DMAP, Pyridine and acetyl chloride, room temperature 2 h. Yield: 100%. **(C)** Chloroacetyl chloride, TEA

and NC043, catalyst DMAP in dry DCM, room temperature for 12 h. **(D)** S-406, biotin, $NaHCO_3$ and catalyst CsCl in DMF, 50°C, 24 h. Final yield: 45%. **(E)** Similar to (c), taking S-1 as a substitute for NC043. **(F)** Similar to (d) with the products from (e) as substitutes for S-406.

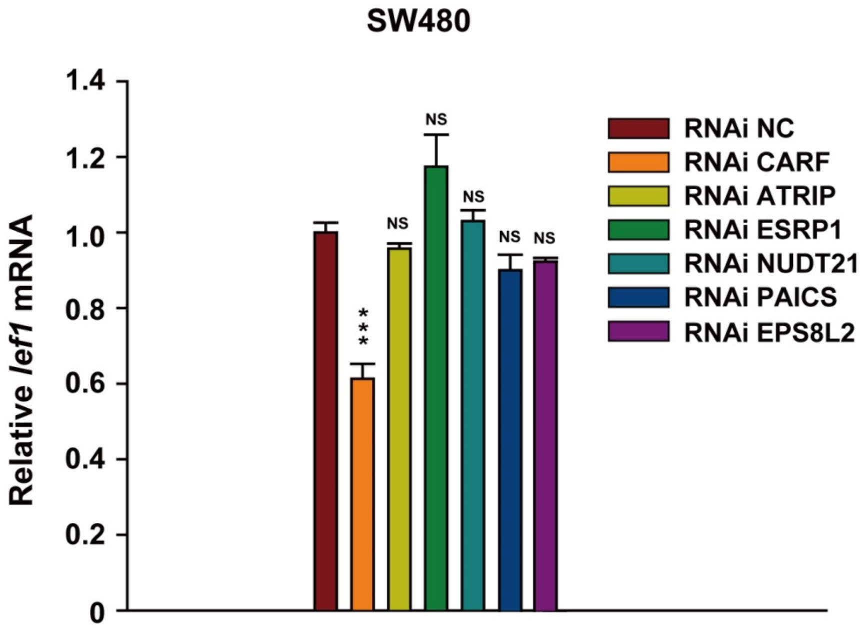

Supplementary Figure S2. Verification of the candidates from MS data with RT-PCR. Of those 8 proteins found in both whole-cell lysate and nuclear extract samples (as shown in Supplementary data set 1), 6 were picked out by excluding the two subunits of condensin complex. We then knockdowned these 6 candidates one by one in SW480 cells via their respective siRNA. After 48 h RNAi, expression of *lef1* was determined by RT-PCR and normalized to GAPDH expression (internal control). The relative expression levels were further normalized with the negative control siRNA (RNAi NC). Error bars indicate the s.d. of triplicate assays in one experiment. Each experiment was repeated at least three times. (* p-value <0.05; ** p-value <0.01; *** p-value <0.001).

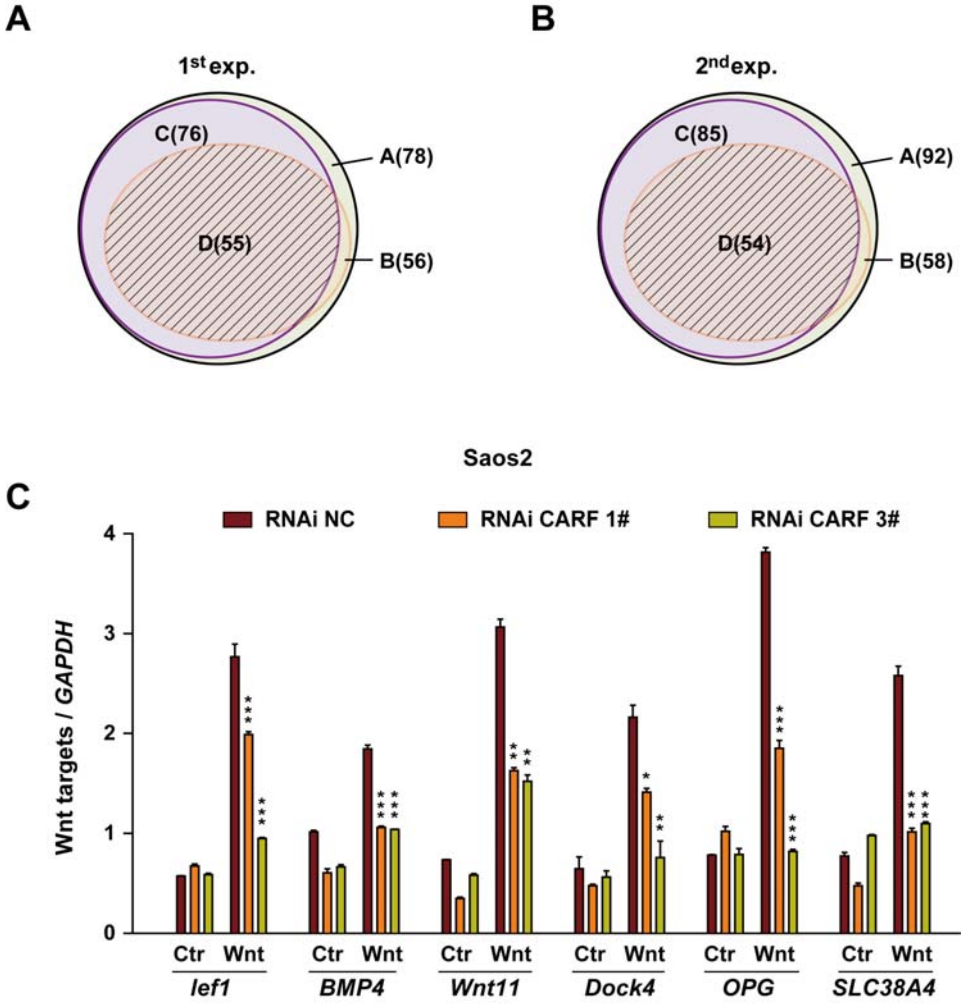

Supplementary Figure S3. Analysis and validation of the microarray data. **(A&B)** Veen diagram sketched the relationship among Wnt-activated genes and CARF- or β-catenin regulated genes. A, Set of genes responsive to Wnt3a stimulation; B, Set of genes with a decreased expression after CARF knockdown; C, Set of genes downregulated by β-catenin knockdown; D, The intersection of B&C. **(A)** & **(B)** represent the results from two independent experiments respectively. **(C)** Validation for microarray data. After knockdown of CARF expression with different CARF siRNAs, 6 genes were picked out randomly for mRNA level detection with q-PCR. All the signals were normalized with those of *GAPDH*. Error bars indicate the s.d. of triplicate assays in one experiment. Each experiment was repeated at least three times. (* p-value <0.05; ** p-value <0.01; *** p-value <0.001).

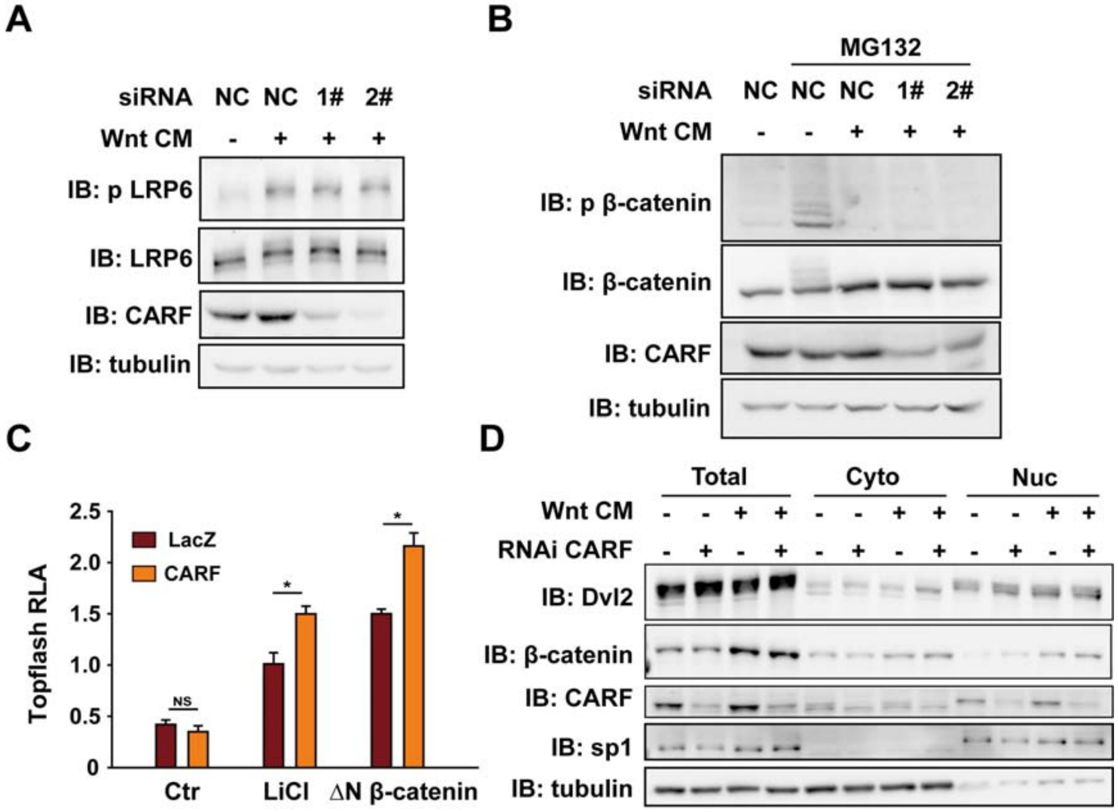

Supplementary Figure S4. CARF functions downstream of β-catenin accumulation. (A) Knockdown of CARF does not affect Wnt-induced LRP6 phosphorylation. After knockdown of CARF for 48 h, HEK293cells were then incubated with control or Wnt3a CM for an additional 30 min and used for western blot analysis. Tubulin was detected as the loading control. **(B)** Knockdown of CARF does not affect β-catenin phosphorylation. CARF-deprived HEK293 cells were incubated with 10 μM MG132. After 1 h, cells were treated with control or Wnt3a supplemented with MG132 for an additional 3 h, followed by western blot assay. Tubulin was used as the loading control. **(C)** Overexpression of CARF further enhances TOPFlash activity induced by LiCl or ΔN β-catenin. For LiCl-induced reporter assay, HEK293 cells were transfected with plasmids as indicated. After 20 h, cells were added with 20 mM LiCl for an additional 6 h and then subjected to reporter assay. For ΔN β-catenin–induced reporter assay, after transfection with ΔN β-catenin and CARF, HEK293 cells were lysed and used for the assay. Error bars indicate the

s.d. of triplicate assays in one experiment. Each experiment was repeated at least three times. (* p-value <0.05; ** p-value <0.01; *** p-value <0.001). **(D)** CARF knockdown does not affect the subcellular localization of β-catenin and Dvl2. After knockdown of CARF for 48 h, HEK293 cells were treated with control or Wnt3a CM for additional 3 h, and then were fractionated and subjected to western blot analysis. Tubulin and SP1 served as the loading controls for the cytosolic and nuclear fractions respectively.

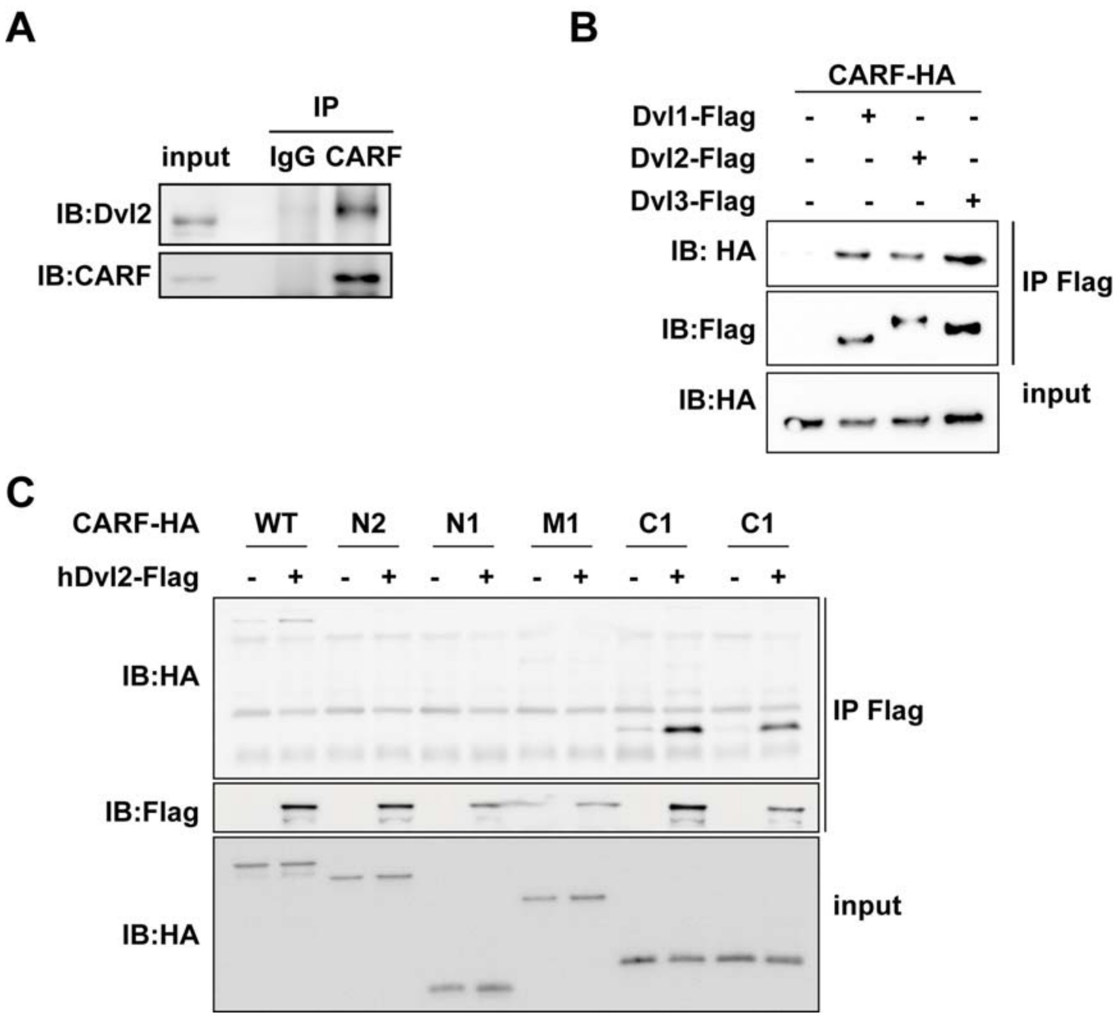

Supplementary Figure S5. CARF interacts with Dvl. (A) CARF interacts with Dvl endogenously. Nuclear extracts of HEK293 cells were used for Co-IP with CARF-specific antibody followed by western blot analysis using anti-Dvl2. IgG was used as a negative control for the IP analysis. **(B)** All the three members of human Dvl family could interact with CARF. HA-tagged CARF and Flag-tagged Dvl1/2/3 were co-transfected into HEK293T cells. 24 h later, cells were harvested and subjected to Co-IP using Flag antibody and western blot analysis with HA antibody. **(C)** Dvl2 binds to the C-terminus of CARF. Dvl2 and the indicated constructs of CARF were co-expressed in HEK293T cells. 24 h later, cells were subjected to Co-IP analysis using Flag antibody followed by western bolt assay with anti-HA antibody.

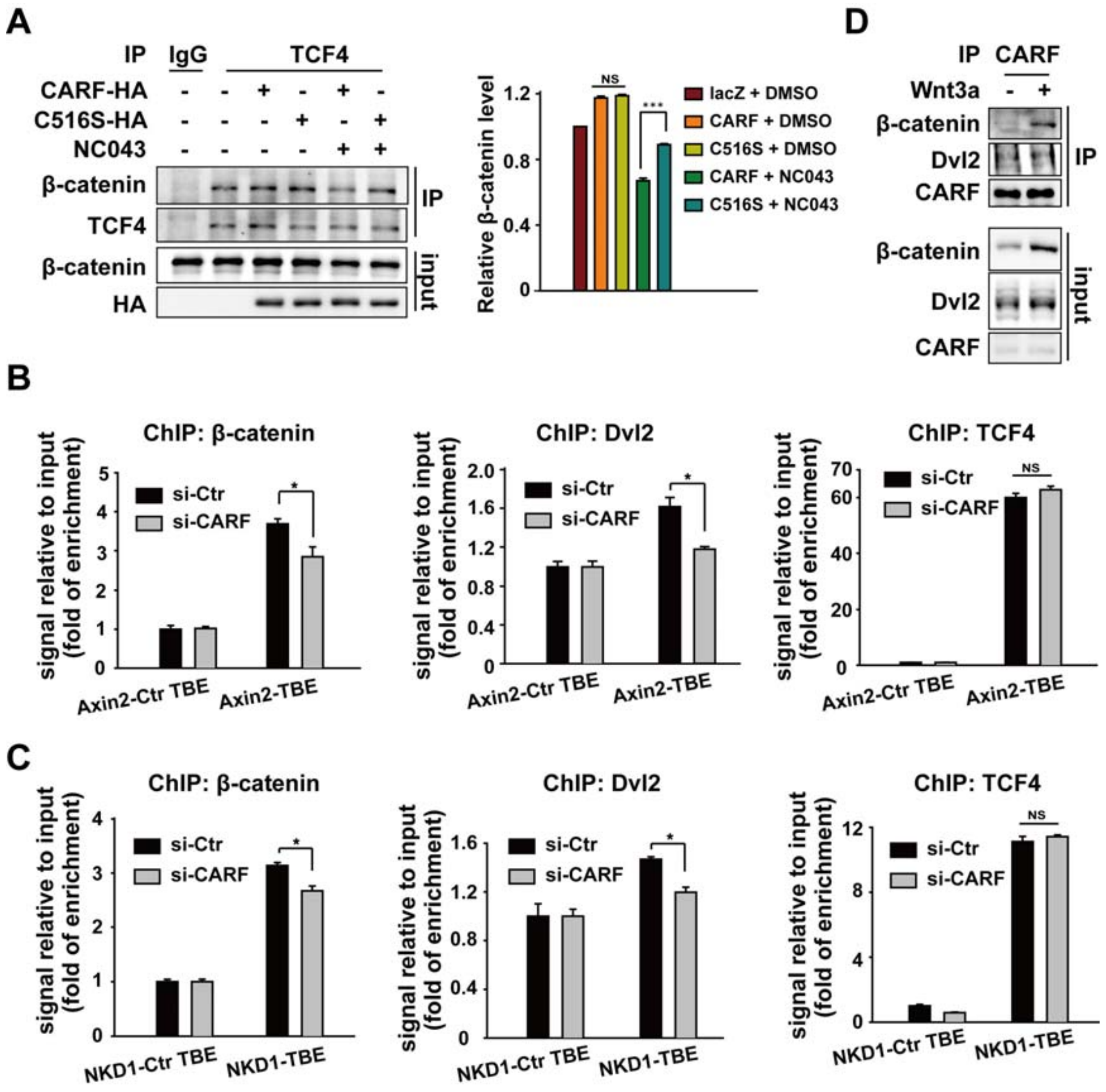

Supplementary Figure S6. The effects of CARF knockdown on the TCF transcriptional complex. **(A)** NC043 decreases the activity of CARF in promoting TCF–β-catenin association. HEK293 cells were transfected with wild-type CARF or its C516S mutant. 15μM NC043 or DMSO was added as indicated 1 h before cell harvest. Nuclear fractions were extracted and then subjected to IP with TCF4-specific antibody and β-catenin was detected with western blot assay. Left panel: results of western blot. Right panel: the levels of β-catenin co-precipitated by TCF4 according to its intensity in western blot analysis. **(B, C)** The effects of CARF on enrichment of β-catenin, Dvl2 and TCF4 on the promoters of *Axin2* and *NKD1*. After knockdown of CARF via siRNA for 48 h, SW480 cells were harvested for chromatin

immuno-precipitation (ChIP) using the antibodies specific to β-catenin, Dvl2 and TCF4. The samples were then subjected to q-PCR assay using primers specific for the indicated proteins. **(D)** The effect of Wnt3a stimulation on interactions of CARF with Dvl2 and β-catenin. After 3 h of control or Wnt3a treatment, HEK293 cell were subjecetd to IP with CARF-specific antibody. Error bars indicate the s.d. of triplicate assays in one experiment. Each experiment was repeated at least three times. (* p-value <0.05; ** p-value <0.01; *** p-value <0.001).

SUPPLEMENTARY METHODS

Cell culture. HEK293T and HEK293 were cultured in DMEM supplemented with 10% (v/v) FBS in a humidified incubator at 37°C and 5% CO_2/95% air (v/v). Saos-2 cells were cultured in McCoy's 5A Medium supplemented with 15% (v/v) FBS. SW480 cells were cultured in Leibovitz's L-15 Medium completed with 10% FBS, at 37°C, without CO_2. All medium and FBS used were purchased from Gibco.

Antibodies. Immunoblotting was performed using the following antibodies: anti-CARF (manufactured by Shanghai Abiocode Biotechnology Co., LTD; dilution 1:2,000), anti-Dvl2 (no. 3224, Cell Signaling Technology, Inc.; dilution 1:1,000); anti–β-catenin (610154, BD Transduction Laboratories; dilution 1:1,000); anti-TCF4 (05-511,Millipore; dilution 1:1,000); anti-sp1 (S9809, Sigma; dilution 1:500); anti–β-actin (sc-47778, Santa Cruz Biotechnology; dilution 1:5,000); anti–β-tubulin (T5168, Sigma; dilution 1:1,000); anti–phospho-β-catenin (Ser33/Ser37/Thr41) (no. 9561P, Cell Signaling Technology, Inc.; dilution 1:1,000); anti-LRP6 (no. 3395, Cell Signaling Technology, Inc.; dilution 1:1,000); anti-phospho-LRP6 (Ser1490) (no. 2568, Cell Signaling Technology, Inc.; dilution 1:1,000); anti-hemagglutinin (1666606, Roche; dilution 1:1,000); anti-Flag (F3165, Sigma; dilution 1:1,000); anti-His6 (H1029, Sigma; dilution 1:1,000); anti-GST (G1160, Sigma; dilution 1:1,000).

Plasmids and siRNAs. TOPFlash and FOPFlash were purchased from Millipore. CARF (GenBank accession number NM_017632.3) cDNA was amplified by PCR from a HEK293T cDNA library and cloned into the mammalian expression vector pCMV-N-HA to generate a N-terminal HA tagged CARF protein (pHA-CARF), and verified by DNA sequencing. The fragments of CARF were amplified by PCR using pHA-CARF as the template and cloned into the pCMV-N-HA vector in a way similar to that of pHA-CARF. CARF point mutants (C503S, C507S, C516S, C545S and CARF*) were generated by site-directed mutagenesis according to the method described in the QuikChange site-directed mutagenesis kit (Stratagene, Palo Alto, CA,

USA). Bacterial expression construct GST-hCARF C1 was obtained by subcloning the CARF C1 fragment into pGEX-4T-1 vector (Novagen) and the bacterial expression construct His-mDvl1 was generated as described previously[1]. The specific primers used in the above studies are listed in **Supplementary Table S1**. siRNAs used were synthesized by GenePharm and their sequences were listed in **Supplementary Table S2**.

Mass spectrometry analysis. The proteins pulled down are lysed by incubation at 95°C for 5 min in 50 µl lysis solution containing 4% SDS, 100 mM Tris/HCl pH 7.6, 0.1 M DTT. The DNA was sheared by sonication to reduce the viscosity of the sample. Before starting sample processing, the lysate was clarified by centrifugation at 16,000×g for 5 min. The protein samples were digested by Filter Aided Sample Preparation (FASP) Method[2].

The tryptic peptides were separated by nanoflow liquid chromatography and analyzed by tandem mass spectrometry (Thermo Electron Finnigan). The LTQ-Orbitrap equipped with an NSI nanospray source (1.7 kV) was operated in data-dependent mode, in which the normalized collision energy was 35%. Full scan was detected in the Orbitrap analyzer (R=60,000 at m/z 300) followed by MS/MS acquisition of the ten most-intense ions in LTQ. Mass calibration used an internal lock mass (m/z 445.120025), the dynamic exclusion repeat count was 2, the repeat duration was 30s, and the exclusion duration window was 180s.

Raw Orbitrap full-scan MS and ion trap MS2 spectra were processed by MaxQuant 1.0.14.10[3]. A composite target-decoy database was created with the program Sequence Reverser from the MaxQuant package. All identified MS/MS spectra were searched against this target/decoy database (IPI HUMAN database, v3.61) using Mascot version 2.1 (Matrix Science). Spectra were initially searched with a mass tolerance of 7 ppm in MS and 0.5 Da in MS/MS and strict trypsin specificity and allowing up to 2 missed-cleavages. Cysteine carbamidomethylation was searched as a fixed modification, whereas N-acetyl protein and oxidized

methionine were searched as variable modification. The resulting Mascot html-output files were loaded into MaxQuant, and fixed the estimated false discovery rate (FDR) of all peptide and protein identifications at maximum 1%[4].

Determination of interaction partners in label-free pulldown is done using Volcano plot[5]. Briefly, plot results comparing the pulldown to a control pulldown used the statistical software R and the provided script QUBIC-LABELFREE.R (Freely available at https://www.Rproject.org). The script plots the results generated by QUBICvalidator. The ratios are plotted against the negative logarithmic P-value of the t-test. Furthermore it adds a curve representing the selected threshold value and SO and colour codes and marks significant binding partners.

ChIP assay. Approximately 1×10^7 SW480 cells, after transfection with control or CARF siRNA for about 48 h, were used to generate one chromatin preparation and divided into 3 parts for TCF4, β-catenin and Dvl immunoprecipitations respectively, according to the instruction of SimpleChIP Enzymatic Chromatin IP Kit (Magnetic Beads) (cell signaling technology). The primer pairs used for *hAxin2* and h*NKD1* were listed in **Supplementary Table S4**. All ChIP experiments were performed three or more times on independent chromatin preparations.

Zebrafish maintenance and manipulation. Wild-type Tubigen (TU) and $tp53^{M214K/M214K}$ mutant[6] zebrafish line were maintained as previously described[7]. To prevent the formation of melanin pigment, the embryos were incubated in egg water containing 0.045% 1-phenyl-2-thiourea (PTU, Sigma) after gastrulation stage. The embryos were collected at the desired stages[8]. The zebrafish facility and study were approved by the Institutional Review Board of the Institute of Health Sciences, Shanghai Institutes of Biological Sciences, Chinese Academy of Sciences (Shanghai, China).

Synthesis and information of NC043 and its analogs.

S-358: Under the protection of N_2, oxalyl chloride ($(COCl)_2$, 0.30 mmol, 28 μl) was injectioned into 10 ml anhydrous dichlormethane (DCM), and stirred at 0 °C, N,

N-dimethylformmide (DMF, 0.30 mmol, 23 μl) was then added dropwise slowly and stirred at 0 °C for 30 min. NC043 (50 mg, 0.15 mmol, dissolved in 5 ml dichloromethane) was injected into the reaction system. The reaction mixture was transferred to room temperature and stirred for 2 h until the starring material was consumed, 20 ml water was added to quench the reaction. The dichloromethane phase was separated, and the water phase was further extracted with 20 ml dichloromethane. The dichloromethane phases were combined, washed with washed with saturated NaCl solution, dried with anhydrous sodium sulfate, concentrated, and subjected to silica gel column chromatography for crude separation to get compound S-358 (50.9 mg, yield: 94%). ^{1}H NMR (400 MHz, $CDCl_3$) δ: 8.05 (1H, s), 5.95 (1H, d, J = 1.4 Hz), 5.43 (1H, dd, J = 4.4, 10.8 Hz), 5.25 (1H, d, J = 1.4 Hz), 4.24 (1H, dd, J = 2.4, 11.6 Hz), 4.12 (1H, d, J = 11.6 Hz), 3.14 (1H, m), 2.81 (1H, brs), 2.16-2.27 (4H, m), 1.85-1.92 (1H, m), 1.60-1.78 (5H, m), 1.53-1.57 (1H, m), 1.33-1.48 (3H, m), 1.18-1.26 (1H, m), 0.95 (3H, s). ^{13}C NMR (100 MHz, $CDCl_3$) δ: 200.6, 173.7, 160.4, 147.3, 117.3, 76.4, 70.6, 48.7, 46.4, 45.7, 44.6, 40.6, 37.3, 35.6, 32.8, 26.9, 25.8, 23.7, 23.4, 20.1, 17.2. HREIMS m/z 358.1781 $[M]^+$ ($C_{21}H_{26}O_5$, calcd. 358.1780).

S-372: NC043 (50.0 mg, 0.15 mmol) and 4-dimethylaminopyridine (DMAP, 0.075, 9.2 mg were placed in a dry reaction bottle, and disolved in 10 ml anhydrous dichloromethane (DCM), with stirring under the protection of N_2. The bottle was placed at 0 °C, pyridine (0.303 mmol, 24 μl) and acetyl chloride (0.18 mmol, 14 μl) were added subsequently. After stirring for 30 min, the reaction mixture was placed at room temperature and stirred for another 2 h. TLC method was used to track the reaction. When the raw material disappeared, 20 ml water was added to quench the reaction. The dichloromethane phase was separated, the water phase was further extracted with 20 ml dichloromethane phases were combined, washer with saturated NaCl solution, dried with anhydrous sodium sulfate, concentrated, and subjected to get S-372 (56.3 mg, yield: 100%). ^{1}H NMR (400 MHz, $CDCl_3$) δ: 5.84 (1H, d, J = 1.6 Hz), 5.22 (1H, dd, J = 4.4, 11.6 Hz), 5.15 (1H, d, J = 1.6 Hz), 4.15 (1H, dd, J = 3.6, 11.6 Hz), 4.13 (1H, d, J = 11.6 Hz), 3.05 (1H, m), 2.72 (1H, t, J = 2.8, 2.8 Hz),

2.05-2.16 (3H, m), 1.92 (3H, s), 1.68-1.82 (1H, m), 1.08-1.68 (11H, m), 0.86 (3H, s). ^{13}C NMR (100 MHz, $CDCl_3$) δ: 200.4, 173.7, 169.9, 147.4, 116.7, 76.2, 70.4, 48.5, 46.1, 45.6, 44.4, 40.4, 37.1, 35.6, 32.6, 26.8, 25.6, 23.6, 23.3, 20.9, 20.0, 17.1. HREIMS *m/z* 372.1930 $[M]^+$ ($C_{22}H_{28}O_5$, calcd. 372.1937).

S-614: Under N_2 chloroacetyl chloride (45 μl, 0.6mmol) followed by triethylamine (TEA, 0.5 ml) was added to a solution of NC043 (40mg, 0.12mmol), catalyst DMAP in dry DCM (10 mL) and the reaction mixture was stirred at 0 C for 30 min, then at ambient temperature for 12 h and then concentrated. The residue was dissolved in ethyl acetate. The solution was washed with 5% citric acid, water and brine. The separated organic layer was dried (Na_2SO_4) and concentrated giving a dark residue. The residue was purified on a silica gel column (Petroleum ether:Acetone = 10:1-5:1) to give S-406 (42 mg); A solution of S-406 (37 mg, 0.091 mol), biotin(111 mg, 0.45 mmol), $NaHCO_3$ (38.3 mg, 0.45 mmol), catalyst CsCl (2.0 mg) in anhydrous DMF (10 ml) was stirred at 50 °C under a nitrogen atmosphere for 24h and then diluted with EtOAc (100 ml) and washed with water (100 ml). The organic layer was separated and dried over anhydrous Na_2SO_4. After evaporation, the crude product was further purified with flash column chromatography (Petroleum ether : Acetone = 5:1-1:1) to afford S-614 as white foam (17 mg, yield: 45%). Compound S-406: ^{1}H NMR (400 MHz, $CDCl_3$) δ: 5.93 (1H, d, J = 1.2 Hz), 5.39 (1H, dd, J = 5.2, 11.2 Hz), 5.25 (1H, d, J = 1.2 Hz), 4.23 (1H, dd, J = 2.4, 12.0 Hz), 4.12 (1H, d, J = 12.0 Hz), 4.03 (2H, dd, J = 14.8, 18.8 Hz), 3.14 (1H, m), 2.82 (1H, brs), 2.18-2.27 (3H, m), 1.86-1.92 (1H, m), 1.62-1.78 (6H, m), 1.53-1.59 (1H, m), 1.34-1.49 (3H, m), 1.16-1.23 (1H, m), 0.96 (3H, s). ^{13}C NMR (100 MHz, $CDCl_3$) δ: 200.4, 173.7, 166.4, 147.2, 117.3, 76.4, 72.7, 48.7, 46.3, 45.9, 44.5, 41.0, 40.6, 37.3, 35.6, 32.8, 26.9, 25.6, 23.7, 23.5, 20.1, 17.2. HREIMS *m/z* 406.1550 $[M]^+$ ($C_{22}H_{27}ClO_5$, calcd 406. 1547). Compound S-614: ^{1}H NMR (400 MHz, $CDCl_3$) δ: 5.93 (1H, d, J = 1.6 Hz), 5.83 (1H, brs), 5.41 (1H, dd, J = 4.4, 11.2 Hz), 5.15 (1H, brs), 4.57 (1H, s), 4.55 (1H, s), 4.51 (1H, m), 4.36 (1H, m), 4.23 (1H, dd, J = 2.0, 12.0 Hz), 4.11 (1H, d, J = 12.0 Hz), 3.10-3.19 (2H, m), 2.92 (1H, dd, J = 4.8, 12.8 Hz), 2.81 (1H, brs), 2.74 (1H, d, J =

12.8 Hz), 2.45 (1H, t, *J* = 7.2 Hz), 2.13-2.24 (4H, m), 1.18-1.89 (19H, m), 0.95 (3H, s). ^{13}C NMR (100 MHz, $CDCl_3$) *δ*: 200.3, 173.9, 172.9, 167.2, 163.4, 147.3, 117.2, 77.2, 76.4, 71.9, 61.7, 60.5, 60.0, 55.4, 48.6, 46.1, 45.6, 44.4, 40.5, 37.2, 35.6, 33.4, 32.8, 28.1, 28.0, 26.9, 25.7, 24.6, 23.6, 23.4, 20.1, 17.2. HREIMS *m/z* 614.2651 $[M]^+$ ($C_{32}H_{42}N_2O_5S$, calcd. 614.2662).

S-616 (a mixture): S-616 could be prepared by the same synthetic method of S-614 based on S-1. Compound S-616: 1H NMR (400 MHz, CDCl3) δ: 5.90 (2H, brs), 5.38 (2H, s), 5.10 (1H, m), 4.99 (2H, m), 4.93 (1H, s), 4.68 (1H, d, *J* = 11.6 Hz), 4.57 (1H, d, J = 11.6 Hz), 4.58 (2H, brs), 4.35 (2H, brs), 4.19 (2H, d, *J* = 12.0 Hz), 4.05 (2H, d, *J* = 11.6 Hz), 3.83 (1H, s), 3.60 (1H, m), 3.15 (1H, brs), 2.90-3.01 (4H, m), 2.72-2.75 (2H, m), 2.36-2.50 (12H, m), 2.16-2.20 (2H, m), 1.15-2.03 (47H, m), 0.92 (3H, s), 0.91 (3H, s). ^{13}C NMR (100 MHz, $CDCl_3$) *δ*: 13C NMR (101 MHz, cdcl3) δ 174.15, 173.98, 173.50, 173.15, 169.26, 167.89, 156.70, 151.04, 109.83, 107.89, 80.37, 78.94, 77.43, 77.20, 76.45, 76.31, 74.48, 61.82, 61.75, 61.48, 61.14, 60.34, 60.17, 55.42, 55.33, 46.98, 46.38, 46.33, 46.29, 45.97, 45.62, 41.95, 41.31, 40.60, 40.52, 40.41, 37.23, 35.52, 35.43, 33.41, 32.91, 32.68, 32.55, 30.35, 28.05, 28.00, 27.66, 27.32, 27.22, 26.91, 24.93, 24.66, 24.46, 24.19, 23.40, 23.36, 20.27, 20.22, 14.09, 13.70. HRESIMS m/z 639.2725 [M+Na]+ ($C_{32}H_{44}N_2O_8S$, calcd. 639.2711).

SUPPLEMENTARY TABLES FOR METHODS

Supplementary Table S1. Primers used for CARF-related constructs

constructs	Forward primers (5'-3'), Reverse primers (5'-3')
pHA CARF	CCAGCTCGAGGGCGCAGGAGGTGTCG<br>ATAAGAATGCGGCCGCTTATAGTAATTCTTGAGGATGTTTTAG
CARF N1	CCAGCTCGAGGGCGCAGGAGGTGTCG<br>ATAAGAATGCGGCCGCTTACCCTCTTTTCTTCACCTTGG
CARF M1	CCAGCTCGAGGATATCGAGTAGCAATGAAGGG<br>ATAAGAATGCGGCCGCTTATAACTTGCAAGAGAATTTGACAG
CARF C1	CCAGCTCGAGAACCAATGAAGATGTGAAACAGA<br>ATAAGAATGCGGCCGCTTATAGTAATTCTTGAGGATGTTTTAG
CARF N2	CCAGCTCGAGGGCGCAGGAGGTGTCG<br>ATAAGAATGCGGCCGCTTATAACTTGCAAGAGAATTTGACAG
C503S	CAAGAAAGTATTGTTTCTGAATTGAGGTGC<br>GCACCTCAATTCAGAAACAATACTTTCTTG
C507S	GTGAATTGAGGTCCAAGTCTGTGT<br>ACACAGACTTGGACCTCAATTCAC
C516S	GGGCACTGGCTCTGGAAAAAGCAAAG<br>CTTTGCTTTTTCCAGAGCCAGTGCCC
C545S	GGTGGTAAAAATATCTAAAAGGAAATACAG<br>CTGTATTTCCTTTTAGATATTTTTACCACC
CARF*	TGTCGGAGTATCTTAGTCAGAACCCGCG<br>CGCGGGTTCTGACTAAGATACTCCGACA

Supplementary Table S2. siRNA sequences for the targeted genes as indicated

Knockdown constructs	siRNA sequence（5'-3'）
si-NC (negative control)	GCGACGAUCUGCCUAAGAU
si-CARF	CGGAGUACCUGAGCCAGAA
si-ATRIP	AAGGUCCACAGAUUAUUAGAU
si-ESRP1	CACAAUGACAGAGUAUUUAAA
si-NUDT21	CUGCACAUAUUACAAAGCCUA
si-PAICS	GTGTCAAGGAAGGATATAA
si-GFPT1	GGAGAGAGTTATCCAACAA
si-CARF 1[#]	GCUCUCAAGAAAGUAUUGU
si-CARF 2[#]	GGUGCAAGUCUGUGUAUUU
si-CARF 3[#]	CGCAGGAGGUGUCGGAGUA

Supplementary Table S3. Primers used for RT-PCR assay

Genes	Orientation	Sequence
GAPDH	Forward (5'-3')	AGGTCGGAGTCAACGGATTTG
	Reverse (5'-3')	TGTAAACCATGTAGTTGAGGTCA
CARF	Forward (5'-3')	GATGAACTGGTTGCCAAGGT
	Reverse (5'-3')	CGAGCTGAGTTCCCACTCTC
Axin2	Forward (5'-3')	AGGCTAGCTGAGGTGT
	Reverse (5'-3')	AGGCTTGGATTGGAGAA
lef1	Forward (5'-3')	TTCAAGGACGAGGGCGAT
	Reverse (5'-3')	TGTACCCGGAATAACTCGAGTAG
NKD1	Forward (5'-3')	GTCAACCACTCCCCAACATC
	Reverse (5'-3')	AATGGTGGTAGCAGCCAGAC
CCND1	Forward (5'-3')	GCTGGCCATGAACTACCTGGA
	Reverse (5'-3')	TCCATTTGCAGCAGCTCCTC
c-Myc	Forward (5'-3')	GGCTCCTGGCAAAAGGTCA
	Reverse (5'-3')	CTGCGTAGTTGTGCTGATGT
BMP4	Forward (5'-3')	ATGATTCCTGGTAACCGAATGC
	Reverse (5'-3')	CCCCGTCTCAGGTATCAAACT
Wnt11	Forward (5'-3')	GCCAATAAACTGATGCGTCTACA
	Reverse (5'-3')	GTATCGGGTCTTGAGGTCAGC
Dock4	Forward (5'-3')	GGCCTGTCATTGGAAATTGGA
	Reverse (5'-3')	GTGAACGTAGCTGGAAGGAAA
OPG	Forward (5'-3')	GCGCTCGTGTTTCTGGACA
	Reverse (5'-3')	GCGCTCGTGTTTCTGGACA
SLC38a4	Forward (5'-3')	GACGGCCTATGCAATTCCTATC
	Reverse (5'-3')	GACGGCCTATGCAATTCCTATC

Supplementary Table S4. Primers used for ChIP assay

Site	Orientation	Sequence
Axin2-Ctr TBE	Forward (5'-3')	GTCGCAGCCATGGGATAATG
	Reverse (5'-3')	ACTTGGTCTTTGTGGGTGGTT
Axin2-TBE	Forward (5'-3')	CTGGAGCCGGCTGCGCTTTGATAA
	Reverse (5'-3')	CGGCCCCGAAATCCATCGCTCTGA
NKD1-Ctr TBE	Forward (5'-3')	GCCCCTCTCAGCCTGATTTT
	Reverse (5'-3')	GTGGCATCACTGACCTCCAA
NKD1-TBE	Forward (5'-3')	GTAAATTGGAGCCGCCGAGT
	Reverse (5'-3')	GTATTGGGACAGAACAGCCCG

Supplementary Table S5. Sequences used in zebrafish relative experiments

CARF gRNA target site	GGCGCGACGAGTCTGCTGCC
CARF genotyping primer F	ATGCTGATTACCAGGGCAAG
CARF genotyping primer R	GCGAAACCAAGCTCTACTCG

SUPPLEMENTARY REFERENCES

1. Qin Y, Li L, Pan W, Wu D. Regulation of phosphatidylinositol kinases and metabolism by Wnt3a and Dvl. *J Biol Chem* 2009; **284**:22544-22548.

2. Wisniewski JR, Zougman A, Nagaraj N, Mann M. Universal sample preparation method for proteome analysis. *Nat Methods* 2009; **6**:359-362.

3. Cox J, Mann M. MaxQuant enables high peptide identification rates, individualized p.p.b.-range mass accuracies and proteome-wide protein quantification. *Nat Biotechnol* 2008; **26**:1367-1372.

4. Olsen JV, Blagoev B, Gnad F *et al.* Global, in vivo, and site-specific phosphorylation dynamics in signaling networks. *Cell* 2006; **127**:635-648.

5. Hubner NC, Bird AW, Cox J *et al.* Quantitative proteomics combined with BAC TransgeneOmics reveals in vivo protein interactions. *J Cell Biol* 2010; **189**:739-754.

6. Berghmans S, Murphey RD, Wienholds E *et al.* tp53 mutant zebrafish develop malignant peripheral nerve sheath tumors. *Proc Natl Acad Sci U S A* 2005; **102**:407-412.

7. Westerfield M. The zebrafish book : a guide for the laboratory use of zebrafish (Brachydanio rerio). Eugene, OR: M. Westerfield 1993.

8. Kimmel CB, Ballard WW, Kimmel SR, Ullmann B, Schilling TF. Stages of embryonic development of the zebrafish. *Dev Dyn* 1995; **203**:253-310.
